# Synthesizing ginsenoside Rh2 in *Saccharomyces cerevisiae* cell factory at high-efficiency

**DOI:** 10.1038/s41421-018-0075-5

**Published:** 2019-01-15

**Authors:** Pingping Wang, Wei Wei, Wei Ye, Xiaodong Li, Wenfang Zhao, Chengshuai Yang, Chaojing Li, Xing Yan, Zhihua Zhou

**Affiliations:** 1 CAS-Key Laboratory of Synthetic Biology, CAS Center for Excellence in Molecular Plant Sciences, Institute of Plant Physiology and Ecology, Shanghai Institutes for Biological Sciences, Chinese Academy of Sciences, 300 Fenglin Rd, Shanghai, 200032 China; 2 University of Chinese Academy of Sciences, Beijing, 100049 China; 3 Bio-Med Big Data Center, CAS Key Laboratory of Computational Biology, CAS-MPG Partner Institute for Computational Biology, Shanghai Institute of Nutrition and Health, Shanghai, 200031 China

**Keywords:** Biological techniques, Molecular biology

## Abstract

Synthetic biology approach has been frequently applied to produce plant rare bioactive compounds in microbial cell factories by fermentation. However, to reach an ideal manufactural efficiency, it is necessary to optimize the microbial cell factories systemically by boosting sufficient carbon flux to the precursor synthesis and tuning the expression level and efficiency of key bioparts related to the synthetic pathway. We previously developed a yeast cell factory to produce ginsenoside Rh2 from glucose. However, the ginsenoside Rh2 yield was too low for commercialization due to the low supply of the ginsenoside aglycone protopanaxadiol (PPD) and poor performance of the key UDP-glycosyltransferase (UGT) (biopart UGTPg45) in the final step of the biosynthetic pathway. In the present study, we constructed a PPD-producing chassis via modular engineering of the mevalonic acid pathway and optimization of P450 expression levels. The new yeast chassis could produce 529.0 mg/L of PPD in shake flasks and 11.02 g/L in 10 L fed-batch fermentation. Based on this high PPD-producing chassis, we established a series of cell factories to produce ginsenoside Rh2, which we optimized by improving the C3–OH glycosylation efficiency. We increased the copy number of UGTPg45, and engineered its promoter to increase expression levels. In addition, we screened for more efficient and compatible UGT bioparts from other plant species and mutants originating from the direct evolution of UGTPg45. Combining all engineered strategies, we built a yeast cell factory with the greatest ginsenoside Rh2 production reported to date, 179.3 mg/L in shake flasks and 2.25 g/L in 10 L fed-batch fermentation. The results set up a successful example for improving yeast cell factories to produce plant rare natural products, especially the glycosylated ones.

## Introduction

High-efficiency production of artemisinic acid via yeast fermentation has become a milestone for the application of synthetic biology to the production of natural plant products^1,2^. Since then, a series of plant natural products have been successfully heterologously produced in microbial cell factories^3,4^, highlighting the potential for metabolic engineering of microbial chassis to provide reliable approaches for obtaining natural plant products on a large scale, especially rare natural products.

Ginsenoside Rh2, derived from *Panax* species, is a promising candidate for cancer prevention and therapy^5–7^. However, the ginsenoside Rh2 accounts for less than 0.01% of dried *Panax ginseng* roots^8^. Commercially available ginsenoside Rh2 is currently produced mainly via the deglycosylation of major protopanaxadiol (PPD)-type ginsenosides isolated from *Panax* spp. using chemical or biotransformation approaches^9–11^. However, such approaches rely heavily on the cultivation of *Panax* plants and the successive cultivation of *Panax* plants is unsustainable, resulting in low productivity and vulnerability to disease outbreaks^12^. A synthetic biology strategy to artificially produce ginsenoside Rh2 from glucose in a metabolically engineered microbial cell factory is expected.

PPD is the common precursor of all dammarane-type ginsenosides, including ginsenoside Rh2. A key enzyme to glycosylate PPD to produce ginsenoside Rh2 is required. A Korean research group reported the isolation of two glycosyltransferase (UGT) genes involved in the biosynthesis of the ginsenosides Rh2 and Rg3^13^. Nearly concurrently, we also characterized two UGTs (UGTPg45 and UGTPg29) taking parts in the biosynthesis of ginsenosides Rh2 and Rg3 independently^14^. In this previous work, we constructed the strain D20RH18 for *de novo* production of ginsenoside Rh2 from glucose based on the PPD-producing chassis strain ZW-PPD-B (chassis 1.0, with a PPD titer of 75.3 mg/L in shake flasks)^14^. However, the ginsenoside Rh2 production of D20RH18 was relatively low (16.95 mg/L in shake flasks), and the yield of total dammarane-type triterpenoids (dammarenediol II [DM], PPD, and ginsenoside Rh2) was also low. Kinetic analysis of the biopart UGTPg45 to produce ginsenoside Rh2 revealed a low catalytic efficiency, only 1/2500 of that of UGTPg29, which further catalyzes ginsenoside Rh2 into Rg3. Therefore, we reasoned that the limited supply of the precursor for dammarane-type triterpenoid synthesis and poor performance of the key UGT biopart were the two key factors hindering the high-efficiency production of ginsenoside Rh2.

Since the first two reports of the ginsenoside Rh2 biosynthetic pathway, several studies have attempted to optimize this glycosylation step by identifying UGT bioparts from microbial sources or by engineering UGTs to catalyze ginsenoside Rh2 formation. UGTs from bacteria often show substrate flexibility and are potential candidates for ginsenoside Rh2 synthesis. UGT109A1 from *Bacillus subtilis* could catalyze the C3–OH and C12–OH glycosylation of DM, PPD, and protopanaxatriol (PPT)^15^. Another UGT from *B. subtilis*, Bs-YjiC, which shares 94% identity with UGT109A1, could also catalyze the C3–OH and C12–OH glycosylation of PPD to yield ginsenoside Rh2 and an unnatural ginsenoside F12 (3-O-β-D-glucopyranosyl-12-O-β-D-glucopyranosyl-20(*S*)-protopanaxadiol)^16^. Coupling this enzyme with a UDP-glucose regeneration system powered by sucrose synthase, an in vitro one-pot ginsenoside synthesis platform was established to produce 0.2 g/L of ginsenoside Rh2 and 3.98 g/L of F12 using PPD as the substrate^16^. Another group built an efficient ginsenoside Rh2 biosynthetic cell factory by repurposing an inherently promiscuous UDP-glycosyltransferase UGT51 from *Saccharomyces cerevisiae*^17^. Based on a structure-guided semi-rational engineering approach, the catalytic efficiency of UGT51 to convert PPD into ginsenoside Rh2 increased by more than 1800-fold. The yeast cell factory with this engineered glycosyltransferase could produce ~300 mg/L of ginsenoside Rh2 in a 5L fed-batch fermentation system. However, there are still many disadvantages of the use of UGT from bacteria or yeast, such as its poor substrate specificity. For example, the engineered UGT51 can also catalyze the C3–OH glycosylation of many native yeast sterols (e.g., ergosterol), and thus produce diverse by-products and waste UGT enzyme activity^17^. Meanwhile, Bs-YjiC exhibits poor site specificity to C3–OH, resulting in lower ginsenoside Rh2 yield (0.2 g/L) compared with the high F12 yield (3.98 g/L)^16^.

Besides, optimization of the PPD-producing chassis may also improve ginsenoside Rh2 yield. In the past several years, much progress has been made in engineering microbes for high-level production of PPD. For example, by integrating the PPD biosynthesis pathway into a yeast chromosome and overexpressing the rate-limiting gene *tHMG1* (truncated HMG-CoA reductase gene) and three key precursor genes, the resulting PPD-producing strain could produce 148.1 mg/L PPD in shake flasks, and the titer reached more than 1 g/L in fed-batch fermentation^18^. Recently, to overcome the poor coupling between *P*. *ginseng* cytochrome P450 and the *Arabidopsis thaliana* cytochrome P450 reductase ATR1, as well as the high DM accumulation in PPD-producing yeast recombinants, the fusion of PPDS and ATR1 encoding genes was introduced and the PPD production increased significantly, reaching 265.7 mg/L PPD in shake flasks and 4.25 g/L in 5 L fed-batch fermentation, which is the highest PPD production ever reported^19,20^.

In this study, we constructed a new PPD-producing chassis strain to improve the conversion of glucose into PPD by overexpressing all mevalonic acid (MVA) pathway genes and optimizing the expression levels of cytochrome P450 enzymes in yeast. In addition, we employed several strategies to optimize the cell factories to produce ginsenoside Rh2 by improving UGTPg45 expression level and activity, including: (1) increasing UGTPg45 expression by increasing its copy number and engineering its promoter and (2) increasing the in vivo activity of UGTPg45 in yeast via protein engineering based on in vivo directed evolution and searching for novel UGTs with higher C3–OH glycosylation efficiencies from other plant species.

## Results

### Design and construction of the PPD-producing chassis 2.0 strain

**Fig. 1:**
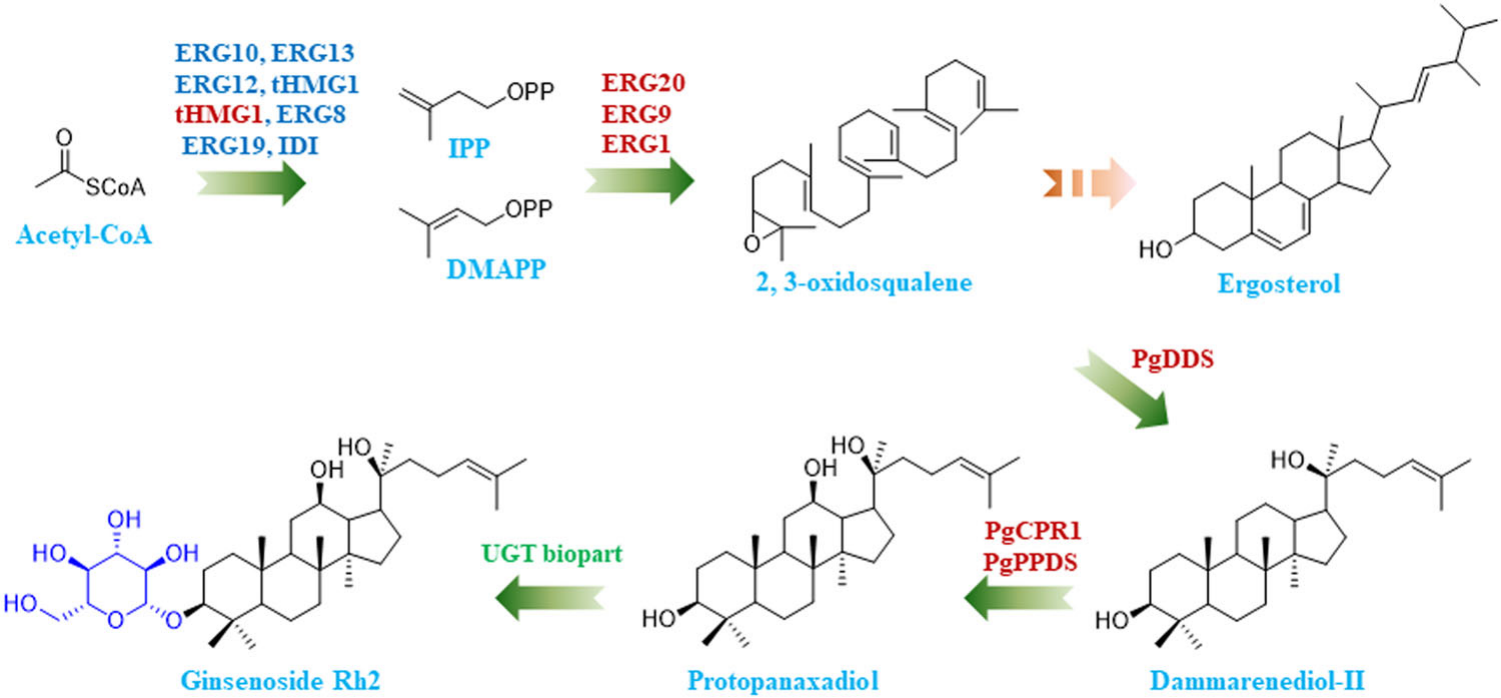
Schematic biosynthesis pathway of ginsenoside Rh2 in engineered yeast. Blue-colored genes form the upstream-module, red-colored genes form the downstream-module, and the green-colored gene is the UDP-glycosyltransferase biopart. IPP isopentenyl pyrophosphate, DMAPP dimethylallyl pyrophosphate

Ten enzymes are required to synthesize the triterpenoid precursor 2, 3-oxidosqualene from acetyl coenzyme A (acetyl-CoA) in the MVA pathway. Then three heterologous genes from *P. ginseng*, which encode three enzymes that convert 2, 3-oxidosqualene into PPD, namely PgDDS, CYP716A47, and PgCPR1, are also required for PPD biosynthesis. Therefore, we aimed to overexpress these 13 genes (Fig. 1 and Supplementary Fig. S1). The 13 genes were divided into two groups. The first group, designated as the upstream-module, included seven upstream genes (*ERG10*, *ERG13*, *ERG12*, *ERG8*, *ERG19*, *IDI*, and *tHMG1*) that converted acetyl-CoA into the isoprenoid building blocks isopentenyl phosphate (IPP) and dimethylallyl phosphate (DMAPP). The second group, designated as the downstream-module, contained the remaining six downstream genes (*ERG1*, *ERG20*, *ERG9*, syn*PgDDS*, syn*PgPPDS*, and syn*PgCPR1*), which converted IPP and DMAPP into the target product PPD. The three heterologous genes *PgDDS*, *CYP716A47*, and *PgCPR1* were codon-optimized for expression in yeast and renamed syn*PgDDS*, syn*PgPPDS*, and syn*PgCPR1*, respectively. Because HMG-CoA reductase catalyzes the rate-limiting step in the MVA pathway, multi-copy overexpression of *tHMG1* benefits the production of different terpenoids^1,21^. Therefore, we added an additional copy of *tHMG1* to the second group (Fig. 1).

**Fig. 2:**
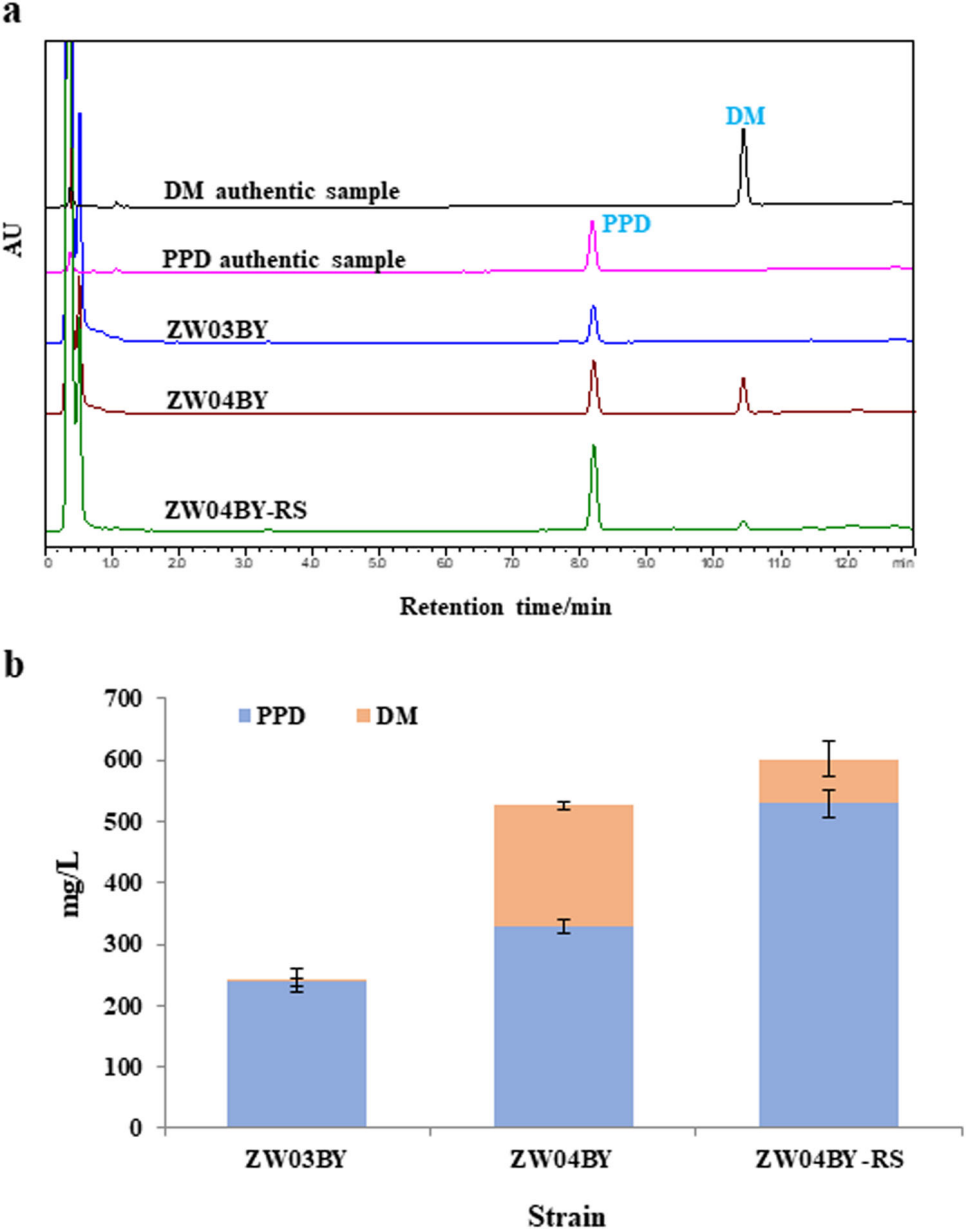
Construction of the protopanaxadiol (PPD)-producing chassis 2.0 strain. **a** High-performance liquid chromatography (HPLC) analysis of dammarenediol II (DM) and PPD produced by ZW03BY, ZW04BY, and ZW04BY-RS. **b** Quantification of DM and PPD produced by ZW03BY, ZW04BY, and ZW04BY-RS. The error bars indicate the SEMs of three biological replicates

After integrating the downstream-module into the *delta DNA* site of the yeast strain BY4742, the resulting strain, ZW03BY, produced 2.7 mg/L DM and 237.9 mg/L PPD from glucose in shake flasks (Fig. 2). This demonstrated that our design should be feasible, because only a single-step integration was required to increase the PPD titer by more than threefold compared with that of ZW-PPD-B (i.e., chassis 1.0). In the next step, the upstream-module was integrated into the *Yprc-delta15* site of ZW03BY, yielding strain ZW04BY. Its PPD titer further increased to 329.7 mg/L in shake flasks. However, the PPD titer of strain ZW04BY was only 39% greater than that of strain ZW03BY, and did not reach the expected target (Fig. 2). Meanwhile, the accumulated DM production in strain ZW04BY reached to 195.9 mg/L (Fig. 2), and the total triterpenoid (PPD + DM) production of strain ZW04BY was 525.6 mg/L, a more than twofold increase compared with that of strain ZW03BY (240.6 mg/L). This indicated that introducing the upstream module boosted the metabolic flux toward the MVA pathway, although additional efforts were required to convert the accumulated DM into PPD.

To further increase the conversion of DM into PPD, another copy of *synPgPPDS* was introduced into the *rDNA* sites of ZW04BY to construct strain ZW04BY-RS, and PPD production reached 529.0 mg/L in shake flasks, which was more than twofold that of strain ZW03BY. In addition, DM production decreased to 72.6 mg/L, representing only 12.1% of the total triterpenoid (PPD + DM) production (Fig. 2).

These results suggest that the increasing expression of key P450 genes could enhance the efficient conversion of DM into PPD. Moreover, the PPD titer of ZW04BY-RS was sixfold that of strain ZW-PPD-B and 1.99-fold that of W3a, the highest PPD production strain constructed in the previous report^19^, representing the highest reported production of PPD in a shake flask system. Therefore, strain ZW04BY-RS might serve as a better chassis to produce ginsenoside Rh2.

### Optimization of UGTPg45 expression in ZW04BY-RS

**Fig. 3:**
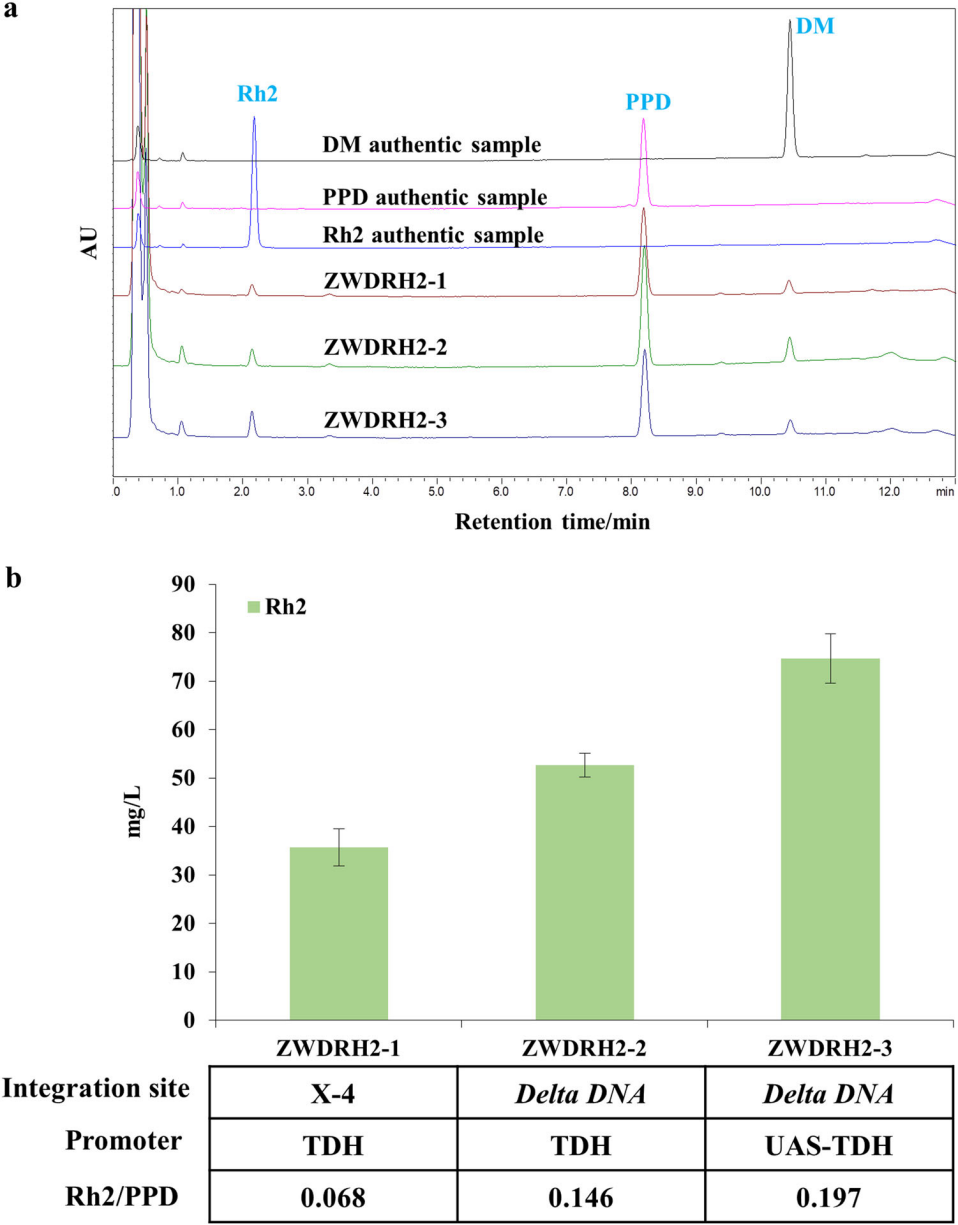
Optimization of UGTPg45 expression in ZW04BY-RS. **a** HPLC analysis of DM, PPD, and ginsenoside Rh2 produced by ZWDRH2-1-3. **b** Quantification of DM, PPD, and ginsenoside Rh2 produced by ZWDRH2-1-3. The error bars indicate the SEMs of three biological replicates

To assess the ginsenoside Rh2 biosynthesis efficiency of the new PPD chassis, UGTPg45 under the control of a strong constitutive promoter, *TDH3*, was introduced into the single-copy X-4 site of ZW04BY-RS^22,23^. However, the resulting strain ZWDRH2-1 produced only 35.7 mg/L of ginsenoside Rh2 in shake flasks (Fig. 3), and the Rh2 yield increased only 110% compared with that of strain D20RH18. In addition, the PPD yield of the newly built strain was 523.2 mg/L. This indicated that only a small amount of PPD was converted into ginsenoside Rh2 in ZWDRH2-1, and its glycosylation ratio (i.e., Rh2/[PPD + Rh2]) was only 6.4%. Therefore, it was necessary to increase the expression level or activity of the glycosyltransferase.

First, we attempted to increase UGTPg45 expression by introducing multi-copy of UGTPg45 into the *delta DNA* sites, which has hundreds of copies in the yeast chromosome. The ginsenoside Rh2 yield of the resulting strain ZWDRH2-2 increased to 52.7 mg/L (Fig. 3). Next, we introduced the strong artificial promoter *UAS*_*TEF1*_*-UAS*_*CIT1*_*-UAS*_*CLB2*_*-TDH3* (hereafter *UAS-TDH*) constructed by Blazeck et al. to replace the *TDH3* promoter^24^, yielding the strain ZWDRH2-3. As a result, the ginsenoside Rh2 yield further increased to 74.7 mg/L (Fig. 3). Overall, ginsenoside Rh2 production was increased by 109% by enhancing UGTPg45 expression level in the PPD chassis ZW04BY-RS.

### Screening for novel UGT bioparts with higher PPD catalysis efficiencies to produce ginsenoside Rh2

As reported in our previous work, the *k*_cat_*/K*_m_ of UGTPg45 for PPD is 0.0018 μM^−1^ s^−1^, only 1/2500 that of UGTPg29 for Rh2 (7.46 μM^−1^ s^−1^), and this poor catalytic activity has limited the biosynthesis of ginsenosides Rh2 and Rg3 in previously constructed strains^14^. As such, UGTs with higher efficiencies are necessary to improve the yield of ginsenoside Rh2. Many studies have demonstrated that some UGTs do not show strict substrate specificity, only regio-specificity^25,26^. The substrate flexibility of these UGTs has been applied in combinatorial biosynthesis and glycol diversification of natural products^27,28^. Because of the poor performance of UGTPg45 from *P*. *ginseng*, we searched for novel alternative UGT bioparts from other plants to more efficiently convert PPD into ginsenoside Rh2. Three UGT genes, *UGT73F3* from *Medicago truncatula*^29^, *UGT73C10* from *Barbarea vulgaris*^30^, and *UGTPn50* from *Panax notoginseng*, were characterized able to produce ginsenoside Rh2 in in vitro reactions by incubating the raw enzyme solution with PPD and UDP-glucose (Supplementary Fig. S2).

**Fig. 4:**
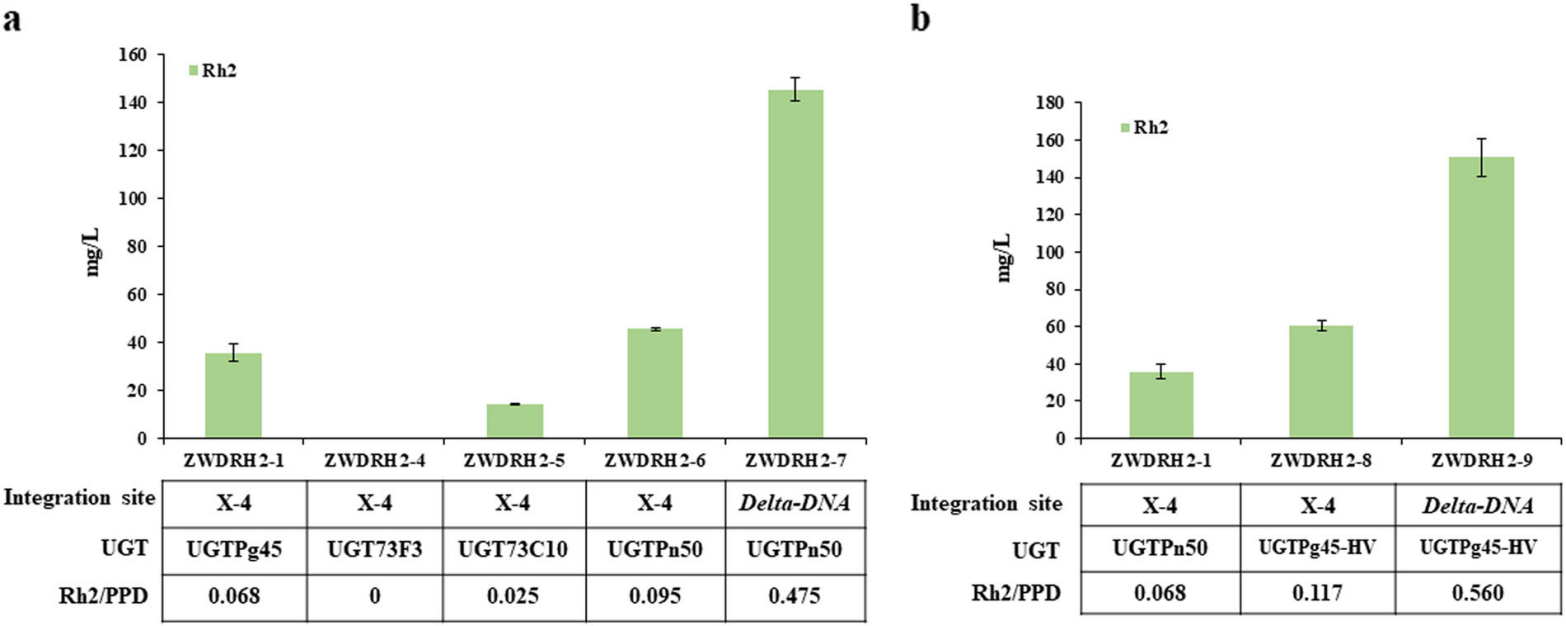
UDP-glycosyltransferase (UGT) biopart optimization for improving ginsenoside Rh2 production. **a** Production of ginsenoside Rh2 and related compounds by novel UGT genes from different sources. UGTPn50, UGT biopart from *P. notoginseng*; UGT73F3, UGT biopart from *M*. *truncatula*; and UGT73C10, UGT biopart from *B. vulgaris*. The UGT biopart UGTPn50, with the highest ginsenoside Rh2 production, was integrated into the multi-copy *delta DNA* sites under the control of the *UAS-TDH3* promoter, yielding strain ZWDRH2-10. **b** Production of ginsenoside Rh2 and related compounds by protein engineering of UGTPg45. The error bars indicate the SEMs of three biological replicates

To compare their ginsenoside Rh2 synthesis efficiencies, the encoding genes *UGT73F3*, *UGT73C10*, and *UGTPn50* were introduced into the PPD-producing chassis ZW04BY-RS at the single-copy X-4 site to construct strains ZWDRH2-4, ZWDRH2-5, and ZWDRH2-6, respectively. ZWDRH2-5 and ZWDRH2-6 produced 14.1 mg/L and 45.6 mg/L of ginsenoside Rh2, respectively (Fig. 4a); however, no Rh2 production was detected in strain ZWDRH2-4 owing to the low enzymatic activity of UGT73F3 toward PPD even in the in vitro test (Supplementary Fig. S2). UGT73C10 can also catalyze the C3–OH glycosylation of DM to produce 3-*O*-glucosyl-dammarenediol II (3-DMG) in vitro, as shown in Supplementary Fig. S3, and the yield of 3-DMG in strain ZWDRH2-5 was 9.3 mg/L.

These results demonstrated that the introduction of UGTPn50 led to a higher ginsenoside Rh2 yield than UGTPg45, resulting in a 27.7% increase in production. Next, we performed the catalytic kinetic analysis of UGTPg45 and UGTPn50. The results demonstrated that the *k*_cat_*/K*_m_ of UGTPn50 (0.127 mM^−1^ s^−1^) was significantly higher than that of UGTPg45 (0.074 mM^−1^ s^−1^) (Table 1). Subsequently, UGTPn50 was integrated into the multi-copy *delta DNA* site of ZW04BY-RS using the strong promoter *UAS-TDH3*, yielding strain ZWDRH2-7. The ginsenoside Rh2 yield of ZWDRH2-7 was 145.7 mg/L in shake flasks, twofold greater than that of ZWDRH2-3 (Fig. 4a).

**Table 1:** The kinetic parameters of engineered and new identified UGT bioparts toward PPD and UDP-glucose as substrates

Enzyme^a^	*V*_max_ (nmol/(min mg))	*K*_m_ (mM)	*k*_cat_ (s^-1^)	*k*_cat_*/K*_m_ (mM^-1^ s^-1^)
UGTPg45	8.69 ± 2.26	0.105 ± 1.06 × 10^–2^	0.0076 ± 2.03 × 10^–3^	0.074 ± 0.027
UGTPg45-HV	15.91 ± 1.07	0.137 ± 5.65 × 10^–2^	0.0139 ± 1.07 × 10^–3^	0.109 ± 0.037
UGTPn50	21.81 ± 2.67	0.152 ± 4.48 × 10^–2^	0.0189 ± 2.70 × 10^–3^	0.127 ± 0.020
UGTPn50-HV	31.71 ± 0.92	0.149 ± 4.04 × 10^–2^	0.0275 ± 1.37 × 10^–3^	0.190 ± 0.042

^a^All given data in this table representing mean values from two or three repeats with corresponding standard deviations

### Screening of UGTPg45 mutants derived from direct evolution with high PPD catalysis efficiencies for ginsenoside Rh2 production

Protein engineering by direct evolution is a powerful strategy for engineering proteins without three-dimensional structures. Thus, an alternative strategy to screen for more efficient UGT was carried out by direct evolution of UGTPg45. Random UGTPg45 mutants in a library constructed with error-prone PCR were integrated into the X-4 site of the PPD chassis ZW04BY-RS. Then, colonies selected from culturing plates were incubated in 96-well plates and subjected to fermentation. The ginsenoside Rh2 yield of each colony extracted with *n*-butanol in the 96-well plates was quantified by HPLC. After optimizing the HPLC running conditions, only 5 min was required per sample, and a 96-well plate could be completed within 8 h.

Among the 16 plates of clones that were screened (> 1500 transformants), we acquired the transformant P8-E7 (renamed strain ZWDRH2-8 hereafter), with a ginsenoside Rh2 yield of 60.5 mg/L, 1.7-fold that of strain ZWDRH2-1 with wild-type UGTPg45 (Fig. 4b). We performed sequence analysis of the UGT gene in strain ZWDRH2-8 and identified two missense mutations (Q222H and A322V), the UGT mutant was renamed UGTPg45-HV. Catalytic kinetic analysis revealed that, although the *K*_m_ value of UGTPg45-HV toward PPD and UDP-glucose remained almost unchanged, the *V*_max_ was nearly doubled and the *k*_cat_*/K*_m_ value increased by more than 40% compared with that of UGTPg45 (Table 1). The significant improvement in catalytic activity may explain the increase of ginsenoside Rh2 production in strain ZWDRH2-8 (Fig. 4b).

In order to explore the molecular mechanism for the catalytic efficiency promotion of the mutant UGTPg45-HV, the wild-type UGTPg45 was analyzed under homology modeling and ligand docking. The analyzing data demonstrated that the two mutated sites on UGTPg45-HV were not included in the binding pocket, in which the enzyme directly contact with the substrates PPD or UDP-glucose (Supplementary Fig. S4). Thus, molecular dynamics (MD) simulations were further performed on UGTPg45 and UGTPg45-HV, respectively. The root mean square deviation (RMSD) showed 60 ns was sufficient for both UDP-glucose and PPD to come into stability (Supplementary Fig. S5a). Although the two mutated sites of UGTPg45-HV were supposed to be outside the substrate-binding pocket (Supplementary Fig. S4), the result of MD simulation did show that the two mutated amino acids could significantly affect the binding affinities between the enzyme and the substrate PPD. Calculated binding-free energy of PPD in UGTPg45-HV was −45.22 ± 3.45 kcal/mol, which was much lower than that in UGTPg45 (−33.29 ± 3.54 kcal/mol). This means the two mutated amino acids make UGTPg45-HV binding more tightly with substrate PPD than UGTpg45. Decompositions on the two total free energies (Supplementary Fig. S6) showed the substrate PPD was binding differently in the pocket, which could also be reflected in the averaged structures during the last 10 ns trajectories (Supplementary Fig. S5b–d). These analyses indicate the two mutated amino acids of UGTPg45-HV outside the pocket may alter the configuration of its substrate binding pocket. The above data may explain the higher catalyzing capacity of the mutant enzyme UGTPg45-HV.

UGTPg45-HV was then integrated into the multi-copy *delta DNA* sites under the control of the stronger *UAS-TDH3* promoter to construct strain ZWDRH2-9. The ginsenoside Rh2 yield of ZWDRH2-9 increased to 150.9 mg/L, more than threefold that of ZWDRH2-1 (Fig. 4b).

### Combining the engineered UGTPg45-HV and novel biopart UGTPn50 to increase ginsenoside Rh2 production

**Fig. 5:**
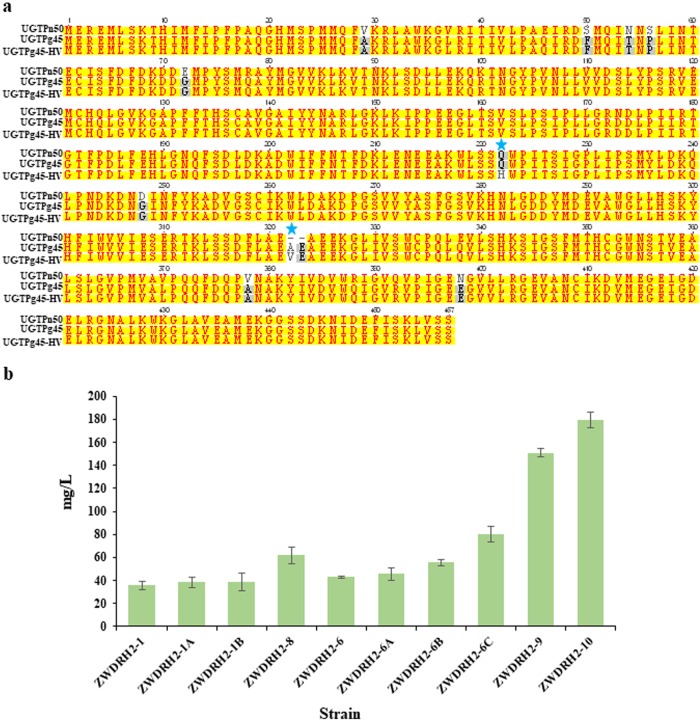
Combination of the engineered UGTPg45-HV and novel biopart UGTPn50 to further improve ginsenoside Rh2 production. **a** Amino acid sequence alignment of UGTPn50, UGTPg45, and UGTPg45-HV. **b** Production of ginsenoside Rh2 by different UGT bioparts in the PPD chassis strain. The error bars indicate the SEMs of three biological replicates

The encoding gene of UGTPn50 from *P*. *notoginseng* shared a high sequence identity with that of UGTPg45. Compared with UGTPg45, two amino acids were missing in UGTPn50, which corresponded to A322 and E323 of UGTPg45 (Fig. 5a). Because the two amino acid mutations Q222H and A322V contributed to the increased activity of UGTPg45-HV, we are wondering whether these two amino acids mutations will also improve the performance of UGTPn50. Thus, the A322V mutation and the other missing amino acid E in UGTPn50 were inserted between E321 and A322 of UGTPn50, yielding the UGT mutant UGTPn50-VE. Moreover, we constructed the single mutant UGTPn50-Q222H and a combined mutant UGTPn50-HV (UGTPn50-Q222H-VE). Then, these three mutants were each integrated into the chromosome of ZW04BY-RS. The resulting strains ZWDRH2-6A, ZWDRH2-6B, and ZWDRH2-6C produced 45.5, 55.6, and 80.0 mg/L of ginsenoside Rh2, respectively (Fig. 5b). Of these, the ginsenoside Rh2 production of strain ZWDRH2-6C was 75.4% greater than that of strain ZWDRH2-6 with wild-type UGTPn50 as the UGT biopart. Therefore, the Q222H mutation and two amino acids VE insertion significantly improved the performance of UGTPn50 in vivo. Ginsenoside Rh2 production was much higher in ZWDRH2-6C than in the strains with the two mutant UGT bioparts (ZWDRH2-6A and ZWDRH2-6B). The same trend was found when comparing ginsenoside Rh2 production of the two single mutations of UGTPg45, UGTPg45-Q222H (strain ZWDRH2-1A) and UGTPg45-A322V (strain ZWDRH2-1B) with UGTPg45-HV (strain ZWDRH2-8) (Fig. 5b). These results suggest that there is a synergistic effect between these two amino acid mutations in improving ginsenoside Rh2 production.

Combining these results, we acquired the improved UGT biopart UGTPn50-HV, and catalytic kinetic analysis revealed that both the *V*_max_ and *k*_cat_*/K*_m_ of UGTPn50-HV increased by more than 40% compared with UGTPn50 (Table 1). Therefore, the final Rh2 cell factory ZWDRH2-10 was constructed by integrating UGTPn50-HV into the *delta DNA* sites under the control of the *UAS-TDH3* promoter. Rh2 production by ZWDRH2-10 reached 179.3 mg/L in shake flasks, which was fivefold that of strain ZWDRH2-1 (Table 2). To the best of our knowledge, this is the highest Rh2 production ever reported in a shake flask system.

**Table 2:** Ginsenosides production by different strains in shake flasks

Strains^a^	Rh2 (mg/L)	PPD (mg/L)	DM (mg/L)
ZW03BY	–	237.9 ± 9.8	2.7 ± 0.5
ZW04BY	–	329.7 ± 4.4	195.9 ± 7.7
ZW04BY-RS	–	529.0 ± 25.5	72.6 ± 2.1
ZWDRH2–1	35.7 ± 3.8	523.2 ± 8.3	67.3 ± 15.7
ZWDRH2-2	52.7 ± 2.5	360.3 ± 13.2	122.3 ± 19.1
ZWDRH2-3	74.7 ± 5.1	375.4 ± 7.4	87.7 ± 9.5
ZWDRH2-4	0	563.4 ± 21.0	88.0 ± 7.4
ZWDRH2-5	14.1 ± 0.1	557.6 ± 48.9	56.7 ± 4.7
ZWDRH2-6	45.6 ± 0.5	478.6 ± 19.4	56.4 ± 4.1
ZWDRH2-7	145.7 ± 4.7	306.7 ± 0.4	184.5 ± 10.7
ZWDRH2-8	60.5 ± 2.5	514.9 ± 2.6	55.8 ± 1.5
ZWDRH2-9	150.9 ± 10.0	269.7 ± 14.6	190.9 ± 48.9
ZWDRH2-1A	38.4 ± 4.2	500.6 ± 5.9	77.3 ± 9.7
ZWDRH2-1B	38.7 ± 7.7	515.2 ± 7.2	65.5 ± 1.3
ZWDRH2-6A	45.6 ± 5.2	475.3 ± 11.0	90.3 ± 20.7
ZWDRH2-6B	55.6 ± 2.5	369.3 ± 1.9	109.0 ± 9.1
ZWDRH2-6C	80.0 ± 6.6	303.3 ± 16.3	174.3 ± 8.8
ZWDRH2-10	179.3 ± 6.5	255.3 ± 19.0	195.3 ± 21.8

^a^All given data in this table representing mean values from three repeats with corresponding standard deviations

### Fed-batch fermentation enabled gram-scale production of PPD and ginsenoside Rh2

Fed-batch fermentation can promote high cell densities and has been proven to boost the production of many valuable natural products, such as artemisinic acid, tanshinone, and ginsenosides, in engineered yeast^1,18,31^. We first performed fed-batch fermentation of the PPD-producing chassis ZW04BY-RS in a 10 L bioreactor. The fermentation was completed within 144 h, and the maximum biomass reached 143.0 g/L dry cell weight (dcw). The final DM and PPD titers reached 7.1 g/L and 11.02 g/L, respectively (Table 3), representing the highest PPD production ever reported. The dry weight content of PPD was 76.9 mg/g dcw (i.e., 7.7% of the dry weight of yeast), much higher than the total saponin content of 5-year-old ginseng root (2– 3%)^8^.

**Table 3:** Ginsenosides production by different strains in 10 L bioreactor

Strains	Biomass (g/L DCW)	Rh2 (mg/L)	PPD (mg/L)	DM (mg/L)
ZW04BY-RS	143.0	–	11017.2	7103.4
ZWDRH2-10	157.3	2252.3	9054.5	8088.8

For fed-batch fermentation of the ginsenoside Rh2 cell factory strain ZWDRH2-10, the fermentation parameters were controlled as the same for ZW04BY-RS. Fermentation was completed within 144 h, and the final biomass was 157.3 g/L dcw. The final DM, PPD, and ginsenoside Rh2 titers were 8.10, 9.05, and 2.25 g/L, respectively (Table 3). This is the highest production and first report of gram-scale production of ginsenoside Rh2, nearly sevenfold that of ZY-7 reported by Zhuang et al^17^.

## Discussion

Due to the complexity of living systems, the developing of a potent artificial biological system with desired function and efficiency is challenging^32^. In the field of natural products production by synthetic biological approaches, many hurdles have to be overcome in order to acquire an efficient cell factory. Such as the developing of a robust chassis strain for sufficient precursor supply, and the characterizing and tuning the compatibilities among different bioparts as well as the compatibilities between key bioparts and the chassis strain^33^.

To build a robust chassis cell with high-level precursor supply, it is necessary to trap more metabolic flux to the related target pathway, modulate the flux distribution between the native and heterogeneous pathway and optimize the rate-limited enzymes. In this study, in order to construct a high-level PPD-producing yeast strain as the chassis cell for the efficient production of ginsenoside Rh2, we aimed to systemically enhance the biosynthetic flux of PPD via overexpressing all the 10 pathway genes. We divided the pathway into two modules and adopted the modular engineering strategies for PPD chassis strain construction^34,35^. A single-step integration of the downstream-module resulted in the production of more than 200 mg/L of PPD in shake flasks (Fig. 2), which demonstrated the feasibility of modular engineering. The subsequent integration of the upstream-module resulted in more than double of total triterpenoid (PPD + DM) production (Fig. 2). Finally, by increasing the expression level of the rate-limited enzyme P450 to convert DM into PPD, the PPD titer reached to more than > 500 mg/L in shake flasks and > 10 g/L in fed-batch fermentation (Fig. 2 and Table 3), which is the highest PPD production and about 2.5-fold of the previously reported highest one^20^.

Due to the long-term evolution, some natural bioparts isolated from native host may suffer from poor expression level, low activities, and other undetectable problems in the microbial chassis cells, which is referred to as biopart-incompatibility^32^. For ginsenoside Rh2 cell factories, the incompatibility of the key UGT biopart UGTPg45 with the PPD-producing chassis (i.e., poor expression and low glycosylation efficiency) extremely limited the final ginsenoside Rh2 yield. Besides, the introducing of UGTPg45 into the PPD-producing yeast chassis dramatically reduced its total triterpenoids production^14^. To screen alternative bioparts with higher compatibility from other source has usually been applied in the optimization of cell factory^21,36^. In fact, some UGTs from bacteria (e.g., UGT109A1 and Bs-YjiC) and yeast (UGT51) have been adopted to produce ginsenoside Rh2^15–17^. The original substrates of those UGTs might be quite different from PPD, however, owing to their lower substrate specificity and even lower regio-specificity, those UGTs turn to be potential bioparts for ginsenoside Rh2 synthesis. Nevertheless, the substrate flexibility or low regio-specificity of those UGTs from microbes often resulted in the production of various by-products^16,17^. Some plant UGTs exhibit lower substrate specificity but high regio-specificity, i.e., these UGTs might catalyze the glycosylation of different aglycons with similar structure at the same position. For example, four UGTs, UGT73C10, UGT73C11, UGT73C12 and UGT73C13, from *B. vulgaris* could glucosylate the C3–OH of different triterpenoids including oleanolic acid, hederagenin, and betulinic acid^30^. Those plant UGTs catalyzing the C3–OH glycosylation of triterpenoids with high regio-specificity provide potential alternative bioparts for ginsenoside Rh2 synthesis. Besides, *P. notoginseng*, another ginsenoside producing plant, may also provide potential UGT bioparts for ginsenoside Rh2 synthesis^37^. In this work, three UGTs, UGT73F3 from *M. truncatula*^29^, UGT73C10 from *B. vulgaris*^30^, and UGTPn50 from *P. notoginseng* were characterized to catalyze the C3–OH glycosylation of PPD yielding ginsenoside Rh2 for the first time (Fig. 4b). UGTPn50 and UGTPg45 belong to UGT74 subfamily, while UGT73F3 and UGT73C10 belong to UGT73 subfamily. The sequence similarity of UGT73F3 and UGT73C10 with UGTPg45 are both less than 30%. The results indicated that UGTs with low sequence similarity might share similar functions. When introducing these three UGTs into the PPD chassis, UGT73F3 with the lowest bioactivity resulted in undetected ginsenoside Rh2 production, UGTPn50 gave the highest ginsenoside Rh2 yield, more than 25% increase compared with that of UGTPg45. UGT73C10, resulted in a medium ginsenoside Rh2 yield (14.1 mg/L) (Fig. 4a) and the production of a by-product 3-DMG (9.3 mg/L). It seems even a plant UGT with low substrate specificity but high regio-specificity to the C3–OH of triterpenoids facing the problems of activities or by-products production in microbial cell factories.

Protein engineering provides another approach to improve the compatibility of bioparts with chassis cells. In this study, we adopt the in vivo directed evolution strategy for the protein engineering of UGTPg45. Compared with previous commonly used in vitro directed evolution methods, in which the screened and selected mutants were then transformed into the chassis, the superiority of an in vivo directed evolution is obvious^38,39^. Because the mutants are introduced directly into the chassis and screened according to the yield of a target product in vivo, the selected mutated bioparts could be improved with not only better enzymatic characteristics but also better performance in compatibility with chassis. In this study, a UGT mutant UGTPg45-HV with two missense mutations (Q222H and A322V), which gave 70% increase of ginsenoside Rh2 yield compared with UGTPg45, was acquired via in vivo directed evolution (Fig. 4b). The catalytic efficiency (*k*_cat_*/K*_m_) of UGTPg45-HV increased by more than 40%, which may explain the increase in ginsenoside Rh2 production (Table 1).

MD simulation analyses demonstrated the two mutant amino acids outside the binding pocket might affect the performance of UGTPg45 by altering the configuration of its substrate binding pocket. As shown in the illustration of the averaged structures (Supplementary Fig. S5b–d), PPD was binding deeper in the binding pocket of UGTPg45-HV, which meant the pocket was less open in the mutant enzyme. In addition to the stronger binding between UGTPg45-HV and PPD, PPD might get a more favorable pose in the narrower pocket of the mutant and thus leaded to the higher catalytic efficiency of UGTPg45-HV. After the two beneficial mutated sites were introduced into UGTPn50, the optimized biopart UGTPn50-HV also demonstrated better in vivo performance and gave the highest ginsenoside Rh2 production in the same PPD-producing chassis (Fig. 5).

In conclusion, we constructed the PPD-producing chassis strain ZW04BY-RS, which yielded a PPD titer of 529.0 mg/L in a shake flask system and 11.02 g/L in a 10 L bioreactor (Fig. 2 and Table 3). Based on this strain, we developed a ginsenoside Rh2 cell factory with a ginsenoside Rh2 titer of 179.3 mg/L in a shake flask system and 2.25 g/L in a 10 L bioreactor (Fig. 5 and Table 3). To the best of our knowledge, this is not only the highest ginsenoside Rh2 yield in engineered microbes, but also the highest yield of natural products with glycosylation modification. We believe the gram-scale per liter yield of ginsenoside Rh2 by the fermentation of ZWDRH2-10 could lower the Rh2 manufacturing cost greatly compared with the traditional ginseng extraction and biotransformation methods. However, it is necessary to carry out pilot plant tests in larger fermenters before commercial production.

## Materials and methods

### Materials, plasmids, and strains

Authentic ginsenoside samples compound K, ginsenoside Rh2, F2, Rg3, Rd, Rb1, Rb2, and protopanaxadiol (PPD) were purchased from Nanjing Zelang Medical Technology Co., Ltd. (Nanjing, China). Dammarenediol II (DM) was purchased from BioBioPha Co., Ltd. (Kunming, China). Plant materials *P. notoginseng* and *M. truncatula* were kindly provided by Prof. Weiming Cai and Prof. Fang Xie, respectively. Both of them are from the Institute of Plant Physiology and Ecology, SIBS, CAS. *E. coli* strain TOP10 was used for gene cloning, and BL21 (DE3) was used for UGTs heterologous expression. *S. cerevisiae* strain BY4742 (*MATα, his3Δ1, leu2Δ0, lys2Δ0, ura3Δ0*) obtained from EUROSCARF was used as the parent strain for all engineering. Codon-optimized genes *synPgDDS*, *synPgPPDS,* and *synPgCPR1* (Genbank accession nos. ACZ71036.1, AEY75213.1, and AIC73829.1, respectively) were synthesized by Genscript Corporation (Nanjing, China). All the strains used or constructed in this study are listed in Supplementary Table S1 and the primers used for the construction of the plasmids and strains are listed in Supplementary Table S2.

### Cloning, synthesis, and heterologous expression of UDP-glycosyltransferases

The coding sequence of UDP-glycosyltransferase UGT73F3 (Genbank accession no. ACT34898.1) was a PCR-amplified form of *M. truncatula* using primer 73F3-F and 73F3-R and cloned into the pMD18T vector (Takara, Dalian, China). The coding sequence of UGTPn50 was a PCR-amplified form of *P. notoginseng* using primer Pn50-F and Pn50-R and cloned into the pMD18T vector. UGT73C10 (Genbank accession no. AFN26666.1) was synthesized by Genscript Corporation.

Heterologous expression of the UGT genes in *E. coli* was carried out as described previously^14^. Briefly, UGT genes with a C-terminally 6×His-tag was ligated into the pET-28a vector via ClonExpress II One Step Cloning Kit (Vazyme Biotech Co., Ltd, Nanjing, China) and transformed into *E. coli* BL21 (DE3). Protein expression was conducted by 0.2 mM IPTG induction for 18 h at 18 °C. The cells were collected by centrifugation and suspended in 100 mM phosphate buffer (pH 7.5) supplemented with 1 mM PMSF and then disrupted with a French Press (25 kpsi). The cell debris was removed by centrifugation (12,000 g, 20 min), and the supernatant was used for enzymatic assays.

### Enzymatic assays of UDP-glycosyltransferases

Enzymatic assays for glycosyltransferases were performed as described previously^14,40^. Generally, the reaction was carried out in a 100 μL system containing 100 mM phosphate buffer (pH 7.5), 1% Tween-20, 5 mM UDP-glucose, 0.5 mM substrate, and 50 μL of UGT crude enzyme (~ 400 ng/mL) for 2 h in a 35 °C water bath. Reaction products was extracted by adding 100 μL of *n*-butanol, the organic phase was then evaporated and dissolved in methanol for TLC and HPLC analysis.

For the kinetic study of UDP-glycosyltransferases, the His-tagged UGTs in the crude enzyme were firstly quantified by dot bolt as described previously^41^. The purified C-terminal 6×His-tagged UDP-glycosyltransferase OleD (Genbank accession no. ABA42119.2) was diluted serially (8, 12, 14, 16, 18, 32 ng/μL) to make a standard curve for protein quantitative. The reaction mixtures contained 100 mM phosphate buffer (pH 7.5), 1% Tween-20, 0.5 mM PPD, 40-300 μM UDP-glucose, and 60 µl crude enzyme of UGTs (~ 400 ng/mL) in a final volume of 300 μL. The reactions were incubated at 30 °C for 30 min. HPLC analysis was used to quantify the target product in each reaction. The Michaelis–Menten parameters were calculated by Lineweaver–Burk plot. All data are presented as means ± SD of two or three independent repeats.

### Construction of yeast strains

The general procedure for each yeast strain construction was descript as following. First, each promoter, gene, terminator, selection marker, and homologous arm was PCR amplified to give the basic fragments, the adjacent basic fragments all sharing 40–75 bp homologous sequences for recombination or fusion PCR; Second, using the adjacent 2–4 basic fragments as template, fusion PCR was performed to give each fusion fragments. Third, the fusion fragments were purified, quantified, and co-transformed into yeast strain via standard LiAc/ssDNA method, because each adjacent fragment sharing 40–75 bp homologous sequences they will be join together via yeast homologous recombination and integrated into chromosome^42^.

### Yeast cultivation and metabolites extraction

Yeast strains were grown in YPD medium composed of 10 g/L Bacto Yeast Extract (BD Difco, USA), 20 g/L Bacto peptone (BD Difco, USA), and 20 g/L dextrose (Sinopharm Chemical, Shanghai, China). For auxotrophic marker selection yeast clones were grown in SD medium composed of 6.7 g/L Difco Yeast Nitrogen Base without amino acids (BD Difco, USA), 20 g/L dextrose and 2 g/L Drop-out Mix Synthetic minus appropriate amino acids (Sigma-Aldrich, USA). For KanMx marker selection yeast clones were grown in YPD medium containing 200 mg/L G418 sulfate (Sangon Biotech, Shanghai, China).

Shake flask fermentation of engineered yeast strains. Individual clones were inoculated into the YPD medium and cultivated at 30 °C, 250 rpm for 16 h. Aliquots were diluted to an initial OD600 of 0.05 in 10 mL of YPD medium in 50 mL shake flasks and grown at 30 °C, 250 rpm for 96 h. The fermentation broth was extracted with *n*-butanol and used for the analysis of DM, PPD, and ginsenoside Rh2.

Fed-batch fermentation of yeast strain ZW04BY-RS and ZWDRH2-10 were conducted in a 10 L bioreactor (T & J Bioengineering, Shanghai, China). The medium for seed, batch fermentation, and the control of feed rate was conducted as previously described^1,18^. About 0.3 L seed of strains ZW04BY-RS or ZWDRH2-10 was inoculated into 2.7 L of batch medium in the 10-L bioreactor and then started the fermentation. Temperature of the fermentation was set at 30 °C. pH was controlled at 5.0 by addition of ammonia hydroxide. Dissolved O_2_ was controlled above 30% by varying speed of agitation or air flow rate. Feeding rate was controlled by maintaining ethanol < 0.5 g/L.

### In vivo direct evolution of UGTPg45

Random mutagenesis of UGTPg45 (Genbank accession no. AKA44586.1) by error-prone PCR was performed using the GeneMorph II random mutagenesis kit (Agilent Technologies Inc., CA, USA). The PCR reaction condition was set as suggested in the user manual, error rate was controlled to be 1–2 mutations per gene. The UGTPg45 mutants was transformed into PPD-producing yeast ZW04BY-RS to generate a mutant library (detailed procedures to construct strain library are provided in the Supplementary Information). The clones that were picked from plates are incubated into deep 96-well plates, which contained 600 μL of YPD medium and cultivated at 30 °C, 280 rpm for 96 h. At the end of fermentation, 600 μL *n*-butanol was added into each well, and PPD and Rh2 were extracted. The organic phase was transformed into a new 96-well plate for quantification of PPD and Rh2 by HPLC.

### Chemical analysis

TLC analysis. The TLC analysis was performed using silica gel 60 F254 plates (Merck KGaA, Darmstadt, Germany) with chloroform/methanol/water (30/10/1, v/v) as the developing solvent. The spots on the TLC plates were visualized by spraying with 1% (w/v) vanillin in H_2_SO_4_/ethanol (6/100, v/v) followed by heating at 110 °C for 5 min. For identification, a mixture of ginsenoside authentic samples containing compound K, ginsenoside Rh2, F2, Rg3, Rb1, Rb2, and Rd, and PPD of concentration 0.2 mg/mL (dissolved in methanol) was also spotted on each plate.

HPLC analysis. The HPLC analysis was performed on a Shimadzu LC20A system (Shimadzu, Kyoto, Japan) equipped with a LC20ADXR pumper, an auto-sampler and a diode array detector. Chromatographic separation of ginsenoside Rh2, PPD, and DM was carried out at 35 °C on a Shim-pack XR-ODS column (100 mm × 2.0 mm, 2.2 μm, Shimadzu, Kyoto, Japan). The gradient elution system consisted of water (A) and acetonitrile (B). Separation was achieved using the following gradient: 0–2.5 min (60% B), 2.5–10 min (60%–90% B), 10–11 min (90% B), and 11–13 min (60% B), and the flow rate was kept at 0.45 mL/min. For rapid detection products of mutants of UGTPg45, Accucore C18 columns (2.6 μm, 2.1 mm × 100 mm, Thermo Scientific, USA) were used and separation was achieved using the following gradient: 0–1 min (55% B), 1–3.5 min (90% B), and 3.5–5 min (55% B), and the flow rate was kept at 0.45 mL/min. Therefore, each sample can be finished within 5 min, and a 96-well plate samples can be finished within 8 h.

### Molecular dynamics simulations and following analyses

Initial structure of WT UGTPg45 was modeled with I-Tasser web server^43–45^. PyMOL v1.7^46^ was used to generate the mutant UGTPg45-HV. UDP-glucose was placed in the binding pocket in the reference of a previous released crystal structure of the complex of a UGT enzyme and UDP-glucose (PDB code: 2ACW^47^). PPD was docked and refined into the binding pocket with AutoDock Vina v1.1.2^48^. MD simulations and most analysis procedures were conducted using the Amber18 software package^49^. Hydrogen atoms were added using the LEaP module. Counter-ions were used to maintain system neutrality. All systems were solvated in a truncated octahedron box of TIP3P waters with a buffer of 10 Å. The *ff14SB* force field^50^ was used for proteins. The parameters of ligands were created using Antechamber with GAFF as basic force field. All the MD simulations were accelerated with the CUDA version of PMEMD and run on NVIDIA^®^ Tesla^®^ P100. Up to 20000-step, steepest descent minimization was performed to relieve any further structural clash in the solvated systems. This was followed by a 400-ps heating up and a 200-ps equilibration in the NVT ensemble at 298K. The 60 ns’ production runs were simulated in the NPT ensemble at 298K with a time step of 2 fs in the Berendsen’s thermostat and barostat with default settings. Detailed simulation information was listed in Supplementary Table S3. Binding-free energies were calculated with MMPBSA with sampling from the last 10 ns of each trajectory, which got into equilibrium in the production run.

## Electronic supplementary material

Supplementary Information

## Electronic supplementary material

**Supplementary Information** accompanies the paper at (10.1038/s41421-018-0075-5).
