# Author Correction: Synthesizing ginsenoside Rh2 in *Saccharomyces cerevisiae* cell factory at high-efficiency

**DOI:** 10.1038/s41421-020-00234-1

**Published:** 2020-12-07

**Authors:** Pingping Wang, Wei Wei, Wei Ye, Xiaodong Li, Wenfang Zhao, Chengshuai Yang, Chaojing Li, Xing Yan, Zhihua Zhou

**Affiliations:** 1 CAS-Key Laboratory of Synthetic Biology, CAS Center for Excellence in Molecular Plant Sciences, Institute of Plant Physiology and Ecology, Shanghai Institutes for Biological Sciences, Chinese Academy of Sciences, 300 Fenglin Rd, Shanghai, 200032 China; 2 University of Chinese Academy of Sciences, Beijing, 100049 China; 3 Bio-Med Big Data Center, CAS Key Laboratory of Computational Biology, CAS-MPG Partner Institute for Computational Biology, Shanghai Institute of Nutrition and Health, Shanghai, 200031 China

**Keywords:** Biological techniques, Molecular biology

Correction to: *Cell Discovery* (2019) 5:5

10.1038/s41421-018-0075-5 Published online 15 January 2019

Following publication of the original article^1^, an error was identified in the “Acknowledgements” section. The funding number for “the National Mega-project for Innovative Drugs (2017ZX09101003-006-002)” is wrong; the correct one is the National Mega-project for Innovative Drugs (2018ZX09711001-006-002). We are sorry for the mistake.

## References

[1] Wang P (2019). Synthesizing ginsenoside Rh2 in *Saccharomyces cerevisiae* cell factory at high-efficiency. Cell Discov..

