# Supplementary material for: Synthesizing ginsenoside Rh2 in Saccharomyces cerevisiae cell factory at high-efficiency
Source: Cell Discov. 2019 Jan 15;5:5. doi: 10.1038/s41421-018-0075-5 (PMC6331602; doi:10.1038/s41421-018-0075-5)
Supplement: Supplementary file 1 — Supplementary Information [file 41421_2018_75_MOESM1_ESM.pdf]

Supplementary Information for:

Synthesizing ginsenoside Rh2 in *Saccharomyces cerevisiae* cell factory at high-efficiency

Pingping Wang [1], Wei Wei [1], Wei Ye [2,3], Xiaodong Li [1,2], Wenfang Zhao [1], Chengshuai Yang [1,2], Chaojing Li [1,2], Xing Yan [1,*], Zhihua Zhou [1,*]

[1] CAS-Key Laboratory of Synthetic Biology, CAS Center for Excellence in Molecular Plant Sciences, Institute of Plant Physiology and Ecology, Shanghai Institutes for Biological Sciences, Chinese Academy of Sciences, 300 Fenglin Rd, Shanghai 200032, China.

[2] University of Chinese Academy of Sciences, Beijing 100049, China.

[3]Bio-Med Big Data Center, CAS Key Laboratory of Computational Biology, CAS-MPG Partner Institute for Computational Biology, Shanghai Institute of Nutrition and Health, Shanghai 200031, China.

[*]Correspondence authors Zhihua Zhou (e-mail: zhouzhihua@sippe.ac.cn) and Xing Yan (e-mail: yanxing@sibs.ac.cn).

Supplementary Figures

Figure S1. Schematic representation the modular construction of strain ZW03BY and ZW04BY.

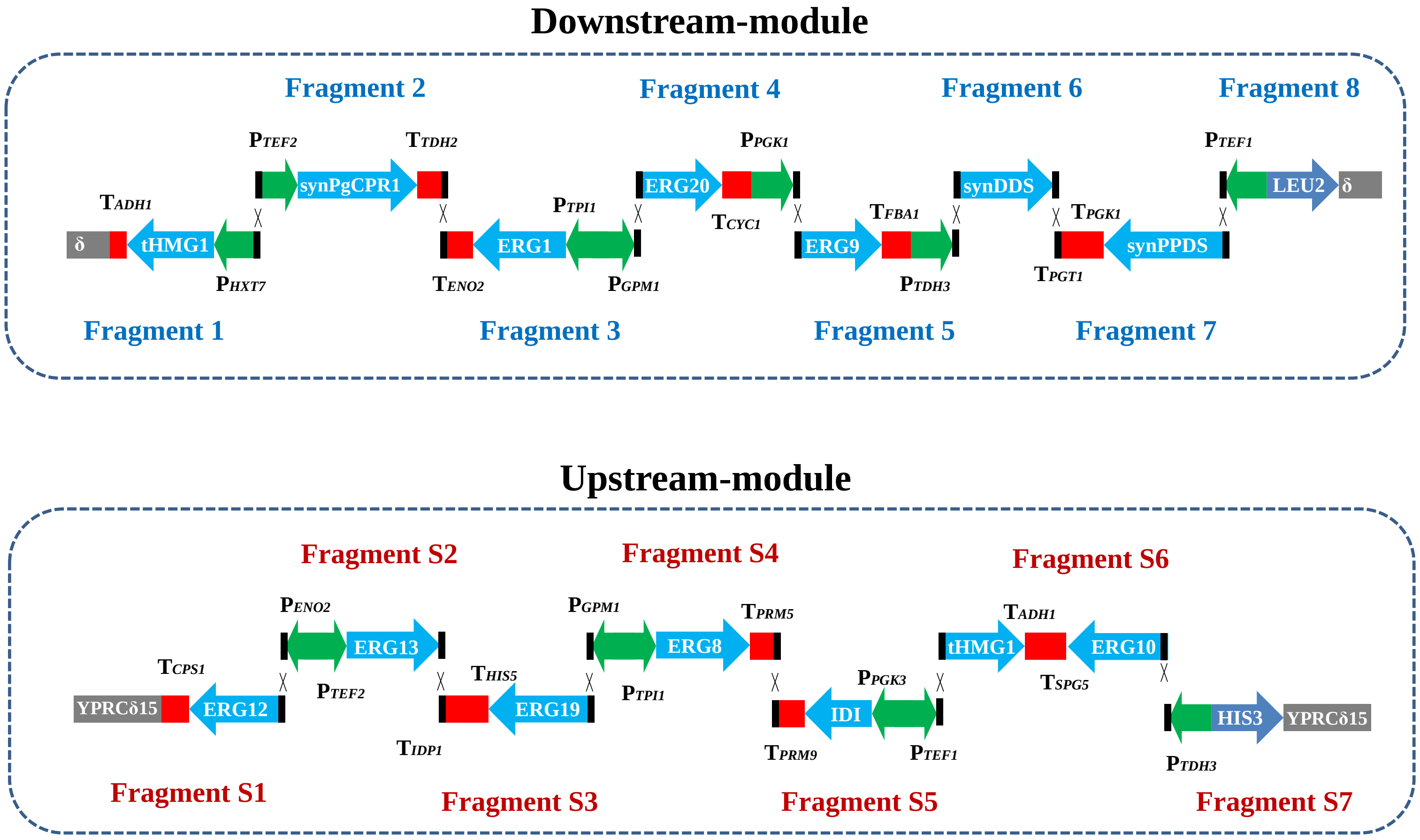

Figure S2. Functional characterization of UGTPn50, UGT73F3, and UGT73C10. Left, thin layer chromatography (TLC) analysis of the reaction product catalyzed by UGTPn50 toward PPD as a substrate; middle, TLC analysis of the reaction product catalyzed by UGT73F3 toward PPD as a substrate; right, TLC analysis of the reaction product catalyzed by UGT73C10 toward PPD as a substrate. M, authentic samples.

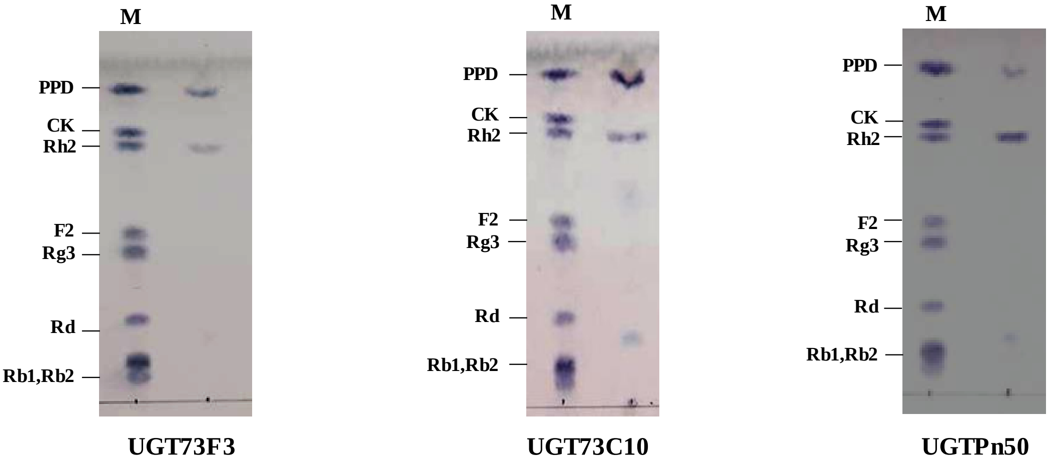

Figure S3. TLC analysis of reaction products catalyzed by UGT73C10 towards DM as substrate.

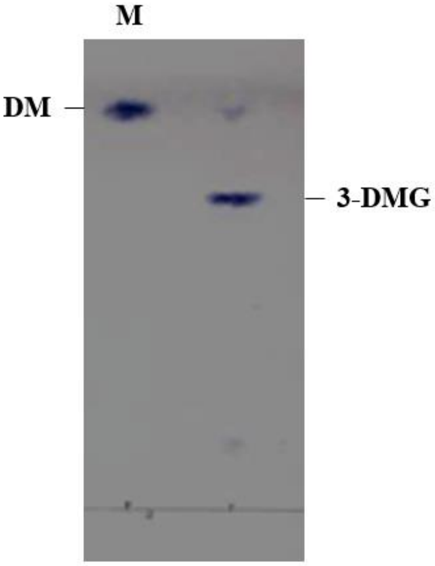

Figure S4. Homology modelling and PPD-docking of wild-type UGTPg45. Two mutated residues in mutant UGTPg45-HV (highlighted in sphere show) are far away from the binding pocket.

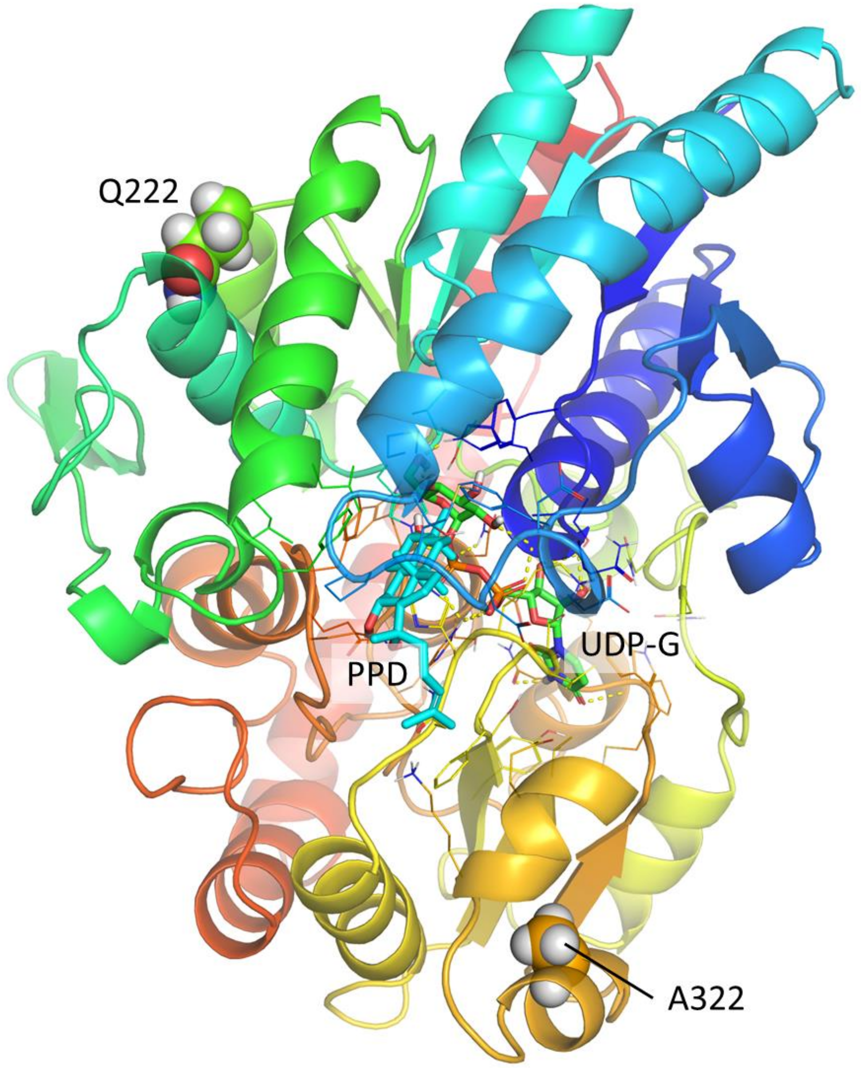

Figure S5. (a) RMSD of substrate PPD along simulation procedures. Blue line for WT UGTPg45, orange line for mutant UGTPg45-HV. (b) Averaged structures of WT (green) and mutant UGTPg45 (yellow), with superimposed together. (c) Surface illustration on the binding pocket for WT UGTPg45. (d) Surface illustration on the binding pocket for mutant UGTPg45-HV.

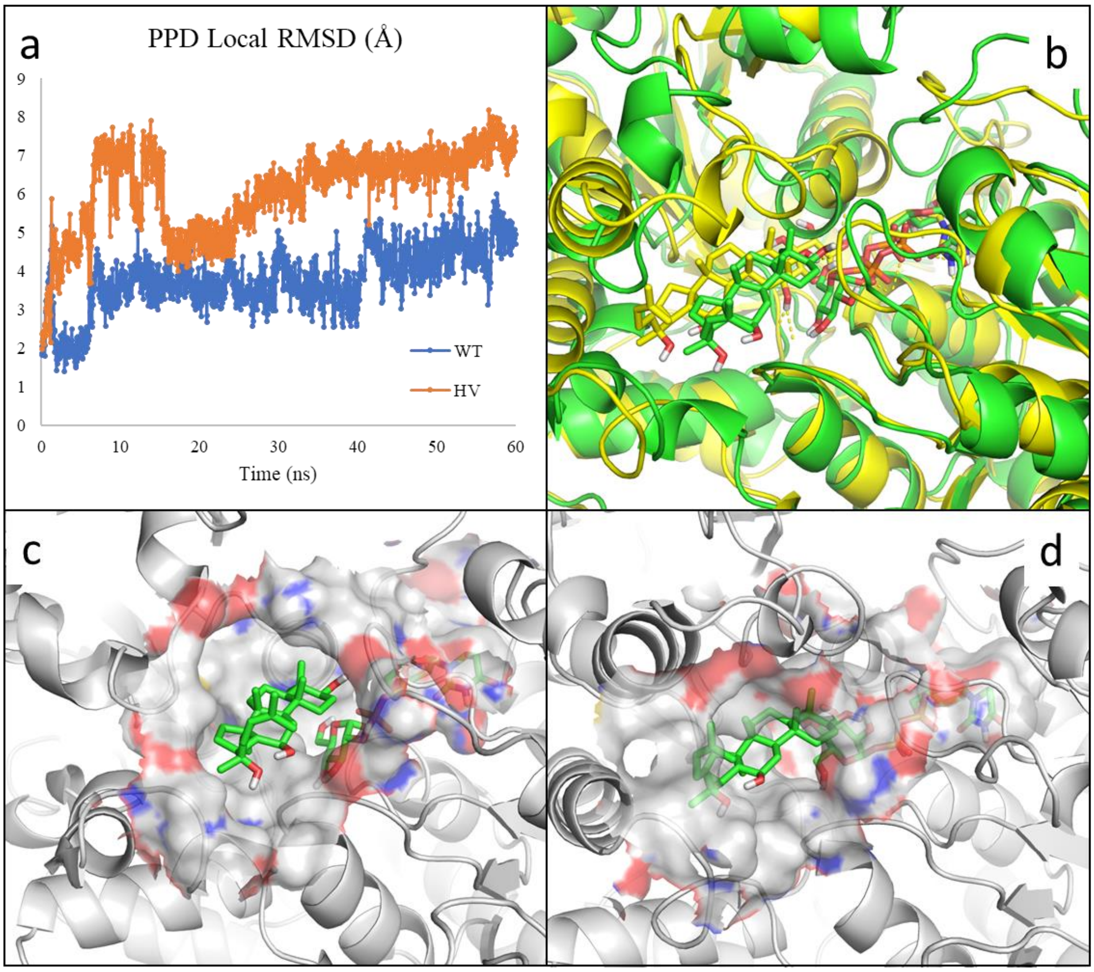

Figure S6. Differences of binding free energy contribution of each residue between WT UGTPg45 and mutant UGTPg45-HV. Positive (red) bars indicate the residues contributing more in WT; Negative (blue) bars indicate the residues contributing more in mutant.

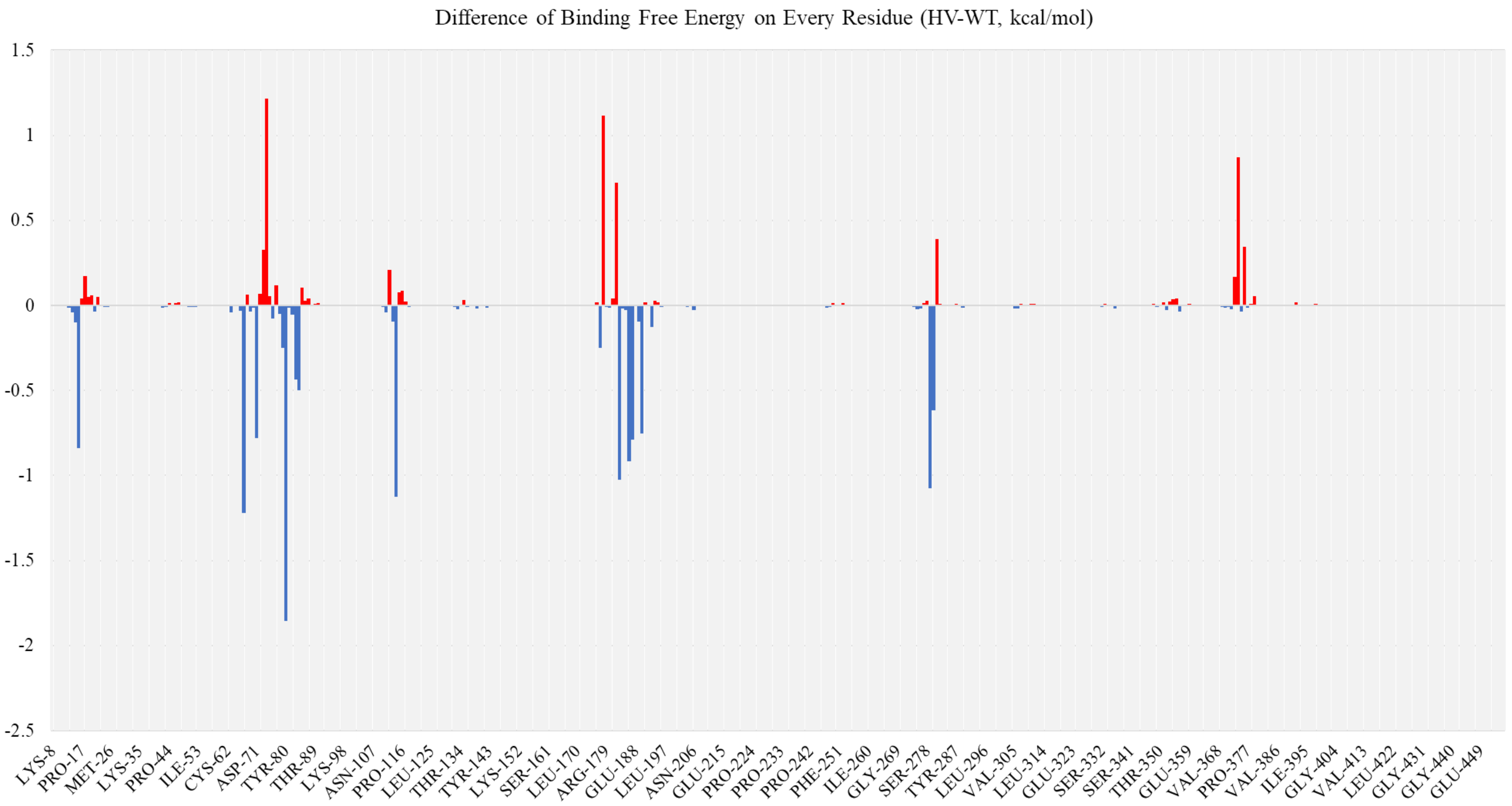

Supplementary Tables

Table S1. Yeast strains used in this study.

Strains	Genotype or characteristic	Source
BY4742	*MAT*α, *his3*Δ1, *leu2*Δ0, *lys2*Δ0, *ura3*Δ0	EUROSCARF
ZW03BY	Downstream module *(HXT7p-tHMG1-ADH1t, TEF2p-synPgCPR1-TDH2t, TPIIp-ERG1-ENO2t, GPM1p-ERG20-CYC1t, PGK1p-ERG9-FBA1t, TDH3p-synDDS-PGT1t, TEF1p-synPPDS-PGK1t,* and *LEU2* marker gene) integrated into δ DNA site of BY4742	This study
ZW04BY	Upstream module *(ENO2p-ERG12-CPS1t, TEF2p-ERG13-IDP1t, TPIIp-ERG8-PRM5t, GPM1p-ERG19-HIS5t, PGK1p-IDI-PRM9t, TDH3p-ERG10-SPG5t, TEF1p-tHMG1-ADH1t,* and *HIS3* marker gene) integrated into *YPRCδ15* DNA site of ZW03BY	This study
ZW04BY-RS	ZW04BY (*rDNA :: TDH3p-synPPDS-CPS1t*)	This study
ZWDRH2-1	ZW04BY-RS (*X-4 :: TDH3p-UGTPg45-PRM9t*)	This study
ZWDRH2-2	ZW04BY-RS (*δ DNA :: TDH3p-UGTPg45-PRM9t*)	This study
ZWDRH2-3	ZW04BY-RS (*δ DNA:: UAS-TDH3p-UGTPg45-PRM9t*)	This study
ZWDRH2-4	ZW04BY-RS (*X-4 :: TDH3p-UGT73F3-PRM9t*)	This study
ZWDRH2-5	ZW04BY-RS (*X-4 :: TDH3p-UGT73C10-PRM9t*)	This study
ZWDRH2-6	ZW04BY-RS (*X-4 :: TDH3p-UGTPn50-PRM9t*)	This study
ZWDRH2-7	ZW04BY-RS (*δ DNA:: UAS-TDH3p-UGTPn50-PRM9t*)	This study
ZWDRH2-8	ZW04BY-RS (*X-4 :: TDH3p-UGTPg45-HV-PRM9t*)	This study
ZWDRH2-9	ZW04BY-RS (*δ DNA:: UAS-TDH3p-UGTPg45-HV-PRM9t*)	This study
ZWDRH2-1A	ZW04BY-RS (*X-4 :: TDH3p-UGTPg45-Q222H-PRM9t*)	This study
ZWDRH2-1B	ZW04BY-RS (*X-4 :: TDH3p-UGTPg45-A322V-PRM9t*)	This study
ZWDRH2-6A	ZW04BY-RS (*X-4 :: TDH3p-UGTPn50-Q222H-PRM9t*)	This study
ZWDRH2-6B	ZW04BY-RS (*X-4 :: TDH3p-UGTPn50-VE-PRM9t*)	This study
ZWDRH2-6C	ZW04BY-RS (*X-4 :: TDH3p-UGTPn50-HV-PRM9t*)	This study
ZWDRH2-10	ZW04BY-RS (*δ DNA:: UAS-TDH3p-UGTPn50-HV-PRM9t*)	This study

Table S2. Primers used for the construction of yeast strains.

Primers	Sequence（5' to 3'）
73F3-F	ATGGAAGGTGTTGAAGTTGAAC
73F3-R	TTAATCATCCAGCTTGAGGTCT
Pn50-F	ATGGAGAGAGAAATGTTGAGCAA
Pn50-R	TCAGGAGGAAACAAGCTTTGAA
03ADH1-F	ACTAGTTAGTAGATGATAGTTGATTTCTATTCCAACAGCATGCCGGTAGAGGTGTGGTC
03ADH1-R	GTTTGAAAGATGGGTCCGTCACCTGCATTAAATCCTAAAGTTATAAAAAAAATAAGTGT
03CPR-F	TTAGAATATACGGTCAACGAACTATAATTAACTAAACATGGCTGCTATGCCTACCTCAT
03CPR-R	AACTAAATCATTAAAGTAACTTAAGGAGTTAAATTTATTACCAGACATCTCTTAAGTAT
03CYC-F	ACTGCGTTCTTGAACAAAGTTTACAAGAGAAGCAAATAGATCCGCTCTAACCGAAAAGGA
03CYC-R	GCAAATGCCTATTATGCAGATGTTATAATATCTGTGCGTCTTCGAGCGTCCCAAAACCTT
03DDS-F	TAATGTTCTTTAGGTATATATTTAAGAGCGATTTGTTTTAAATTTTCAATTGTTGGTGT
03DDS-R	AACTTAGTTTCGAATAAACACACATAAACAAACAAAAATGTGGAAGTTAAAGGTAGCTC
03DN-F	AGGGCGATCGGTGCGGGCCTCTTCGCTATTACGCCAGATGAAGCAGGTGTTGTTGTCTG
03DN-R	TCGAGGAGAACTTCTAGTATATTC
03ENO2-F	GATAAAGCACTTAGTATCACACTAATTGGCTTTTCGCAGGTATCATCTCCATCTCCCAT
03ENO2-R	ATTCACCCCATTTTTGTTTGGTGAGTTGATTGGTTAATAAAGTGCTTTTAACTAAGAAT
03ERG1-F	CAGAAAAGACTAATAATTCTTAGTTAAAAGCACTTTATTAACCAATCAACTCACCAAAC
03ERG1-R	AATCTATAACTACAAAAAACACATACATAAACTAAAAAATGTCTGCTGTTAACGTTGCA
03ERG9-F	GGAAGTAATTATCTACTTTTTACAACAAATATAAAACAATGGGAAAGCTATTACAATTGG
03ERG9-R	ACTCATTAAAAAACTATATCAATTAATTTGAATTAACTTCACGCTCTGTGTAAAGTGTAT
03ERG20-F	TCTTAATAATCCAAACAAACACACATATTACAATAAATGGCTTCAGAAAAAGAAATTAGG
03ERG20-R	TCAGGTTGTCTAACTCCTTCCTTTTCGGTTAGAGCGGATCTATTTGCTTCTCTTGTAAAC
03FBA-F	TTGGGTTTTATTATATATACACTTTACACAGAGCGTGAAGTTAATTCAAATTAATTGATA
03FBA-F	ACTTCTTGTTGTTGACGCTAACATTCAACGCTAGTATAGTAAGCTACTATGAAAGACTT
03GMP1-F	GGTAATCTTCCACCAACCTGATGGGTTCCTAGATATATAGTCGTGCAATGTATGAC
03GMP1-R	CAAGAATCTCTCTCTCCTAATTTCTTTTTCTGAAGCCATTTATTGTAATATGTGTGTTTG
03HMG1-F	ACTTTAAAATTTGTATACACTTATTTTTTTTATAACTTTAGGATTTAATGCAGGTGACG
03HMG1-R	CACAAAAACAAAAAGTTTTTTTAATTTTAATCAAAAAATGGCTGCAGACCAATTGGTGA
03HXT7-F	GGTGACTTCAGTTTTCACCAATTGGTCTGCAGCCATTTTTTTGATTAAAATTAAAAAAAC
03HXT7-R	TACGCTTGACATCTACTATATGTAAGTATACGGCCCCACTTCTCGTAGGAACAATTTCG
03LEU-F	TAAGGCTAACTCTCAACAGACAACAACACCTGCTTCATCTGGCGTAATAGCGAAGAGGC
03LEU-R	AGAAACATTTTGAAGCTATGGTGTGTGGGGGATCACTGCATATACCTTTTTCAACTGAA
03PGK-F	CTGAAAACCTTGCTTGAGAAGGTTTTGGGACGCTCGAAGACGCACAGATATTATAACATC
03PGK-R	GACCGGATGCAATGCCAATTGTAATAGCTTTCCCATTGTTTTATATTTGTTGTAAAAAGT
03PGK1t-F	CCACGGTTTACCTATCCACTTACACCCACACAACTGAGATCTCCCATGTCTCTACTGGT
03PGK1t-R	GCGCGAAGGACATAACTCATGAAGCCTCCAGTATACCAACGCAGAATTTTCGAGTTATT
03PGT-F	GCGTATTTTAAGTTTAATAACTCGAAAATTCTGCGTTGGTATACTGGAGGCTTCATGAG
03PGT-R	AGTTTGGTTACCTAAACACCAACAATTGAAAATTTAAAACAAATCGCTCTTAAATATAT
03PPDS-F	GAAAGCATAGCAATCTAATCTAAGTTTTAATTACAAAATGGCAGCCGCTATGGTTTTGT
03PPDS-R	CAAAGAAGCACCACCACCAGTAGAGACATGGGAGATCTCAGTTGTGTGGGTGTAAGTGG
03TDH-F	TGGGTCATTACCTTGAGCTACCTTTAACTTCCACATTTTTGTTTGTTTATGTGTGTTTA

03TDH-R	TTCGAGTTCTTTGTAAAGTCTTTCATAGTAGCTTACTATACTAGCGTTGAATGTTAGCG
03TDH2-F	GCAAATGACTGGTAGATACTTAAGAGATGTCTGGTAATAAATTTAACTCCTTAAGTTAC
03TDH2-R	CACAGTGATATGCATATGGGAGATGGAGATGATACCTGCGAAAAGCCAATTAGTGTGAT
03TEF1-F	TTTTTCTCCCAATTTTTCAGTTGAAAAAGGTATATGCAGTGATCCCCCACACACCATAG
03TEF1-R	AGGACAATGAAAAGAACAAAACCATAGCGGCTGCCATTTTGTAATTAAAACTTAGATTA
03TEF2-F	GAACACGCAGGGGCCCGAAATTGTTCCTACGAGAAGTGGGGCCGTATACTTACATATAG
03TEF2-R	CAACTAACATAGCCAATGAGGTAGGCATAGCAGCCATGTTTAGTTAATTATAGTTCGTT
03TPI1-F	TTAATCAATTCAGGTGCAACGTTAACAGCAGACATTTTTTAGTTTATGTATGTGTTTT
03TPI1-R	CTGCTCACAAATCTTAAAGTCATACATTGCACGACTATATATCTAGGAACCCATCAGGT
03UP-F	GGATATAGGAATCCTCAAAATG
03UP-R	GAGGTCGCTCTTATTGACCACACCTCTACCGGCATGCTGTTGGAATAGAAATCAACTAT
04ADH1-F	TTTGAAAGATGGGTCCGTCACCTGCATTAAATCCTAAAGTTATAAAAAAAATAAGTGTA
04ADH1-R	GCATAAAAACTTCATGAAAATTCGGCAGAAAATAAGCGCATGCCGGTAGAGGTGTGGTC
04CPS1-F	ACTGAATCTTGTCTGTTTGGTAGCGGCTGCTTTATATTTGACACTTGATTTGACACTTC
04CPS1-R	ATTGCCAGGAAACACGAATTTACCATGGACTTCATAAGCGCAATGATTGAATAGTCAAA
04DN-F	TTTCTTTTTGCTTTTTCTTTTTTTTTCTCTTGAACTCGGCCAGGCGCCTTTATATCATA
04DN-R	GACTATAATATTATGCATATAGGA
04ENO2-F	CCTTTCCCGGTGCAGAAGTTAAGAACGGTAATGACATTTATTATTGTATGTTATAGTAT
04ENO2-R	TACGCTTGACATCTACTATATGTAAGTATACGGCCCCGTGTCGACGCTGCGGGTATAGA
04ERG8-F	ATCTATAACTACAAAAAACACATACATAAACTAAAAAATGTCAGAGTTGAGAGCCTTCA
04ERG8-R	TGCTTAAAAAAAAATATTGCAAAATATCATAAAAGTTTTTATTTATCAAGATAAGTTTCC
04ERG10-F	ACCTTCTTGGTAATAGCGCGATGAAACAACGTCTTTGTCATATCTTTTCAATGACAATA
04ERG10-R	AACTTAGTTTCGAATAAACACACATAAACAAACAAAAATGTCTCAGAACGTTTACATTG
04ERG12-F	TAAAAAAAAAAAAATCTTTGACTATTCAATCATTGCGCTTATGAAGTCCATGGTAAATTC
04ERG12-R	AACACCAAGCAACTAATACTATAACATACAATAATAAATGTCATTACCGTTCTTAACTT
04ERG13-F	TTAGAATATACGGTCAACGAACTATAATTAACTAAACATGAAACTCTCAACTAAACTTT
04ERG13-R	AAAAAAAAAAGTGGTAGATTGGGCTACGTAAATTCGATTATTTTTTAACATCGTAAGAT
04ERG19-F	ATATAAAACTGTACATATACTGTTTAAATTAATCTATTTATTCCTTTGGTAGACCAGTC
04ERG19-R	TTCTTAATAATCCAAACAAACACACATATTACAATAAATGACCGTTTACACAGCATCCG
04GPM-F	TGACGGGTGCGGTAACGGATGCTGTGTAAACGGTCATTTATTGTAATATGTGTGTTTGT
04GPM-R	GGTAATCTTCCACCAACCTGATGGGTTCCTAGATATATAGTCGTGCAATGTATGACTTT
04HIS3-F	ACTTCTTGTTGTTGACGCTAACATTCAACGCTAGTATTCGCGCGTTTCGGTGATGACGG
04HIS3-R	TTTTGTGTCTTAATTATATGATATAAAGGCGCCTGGCCGAGTTCAAGAGAAAAAAAAAG
04HIS5-F	CCTTATCAAGGTTCCCCAAGTTCGGATCATTACCATCGTAACAATATCATGAGACCTTT
04HIS5-R	TTTGATTGACGCAAAGACTGGTCTACCAAAGGAATAAATAGATTAATTTAAACAGTATA
04HMG1-F	GAAAGCATAGCAATCTAATCTAAGTTTTAATTACAAAATGGCTGCAGACCAATTGGTGA
04HMG1-R	ACTTTAAAATTTGTATACACTTATTTTTTTTATAACTTTAGGATTTAATGCAGGTGACG
04IDI-F	CTGGTAAAGTTGTGTGCTAGTGTCTCCCGTCTTCTGTTTATAGCATTCTATGAATTTGC
04IDI-R	GAAGTAATTATCTACTTTTTACAACAAATATAAAACAATGACTGCCGACAACAATAGTA
04IDP1-F	TGACAAATTTAGAAGATCTTACGATGTTAAAAAATAATCGAATTTACGTAGCCCAATCT
04IDP1-R	GGCGCCACTTCTATAAAAGGTCTCATGATATTGTTACGATGGTAATGATCCGAACTTGG
04PGK1-F	GCACCATGGGGCATACTATTGTTGTCGGCAGTCATTGTTTTATATTTGTTGTAAAAAGT
04PGK1-R	AGAAACATTTTGAAGCTATGGTGTGTGGGGGATCACTACGCACAGATATTATAACATCT
04PRM5-F	GAAAGAAAAAGATCCGGAAACTTATCTTGATAAATAAAAACTTTTATGATATTTTGCAA

04PRM5-R	CATTATGCAACGCTTCGGAAAATACGATGTTGAAAATATAGAACCCAAAAAGAGAGACT
04PRM9-F	TTATATTATTTGTTTAGTCTCTCTTTTTGGGTTCTATATTTTCAACATCGTATTTTCCG
04PRM9-R	AGTGGAAAATGACAGGCAAATTCATAGAATGCTATAAACAGAAGACGGGAGACACTAGC
04SPG5-F	GAGGTCGCTCTTATTGACCACACCTCTACCGGCATGCGCTTATTTTCTGCCGAATTTTC
04SPG5-R	TGGTGGTGCTTCCTCTATTGTCATTGAAAAGATATGACAAAGACGTTGTTTCATCGCGC
04TDH3-F	TCTGGCAGTCGATACAATGTAAACGTTCTGAGACATTTTTGTTTGTTTATGTGTGTTTA
04TDH3-R	TGTCAGAGGTTTTCACCGTCATCACCGAAACGCGCGAATACTAGCGTTGAATGTTAGCG
04TEF1-F	AAATGCCTATTATGCAGATGTTATAATATCTGTGCGTAGTGATCCCCCACACACCATAG
04TEF1-R	TGGTGACTTCAGTTTTCACCAATTGGTCTGCAGCCATTTTGTAATTAAAACTTAGATTA
04TEF2-F	GAGTAAAGAACCCTTTCTATACCCGCAGCGTCGACACGGGGCCGTATACTTACATATAG
04TEF2-R	TAATACCACACCAACAAAGTTTAGTTGAGAGTTTCATGTTTAGTTAATTATAGTTCGTT
04TPI-F	CTGCTCACAAATCTTAAAGTCATACATTGCACGACTATATATCTAGGAACCCATCAGGT
04TPI-R	CTTTCCCTGGGGCACTGAAGGCTCTCAACTCTGACATTTTTTAGTTTATGTATGTGTTT
04UP-F	TTTGCCAACAATCGAAACCAAACA
04UP-R	ATAAAAAAAAAAAGAAGTGTCAAATCAAGTGTCAAATATAAAGCAGCCGCTACCAAACA
RSCPS1t-F	CCACGGTTTACCTATCCACTTACACCCACACAACTGAGCGCAATGATTGAATAGTCAAA
RSCPS1t-R	TTGTCGACCTGCAGCGTACGAAGCTTCAGCTGATTTGACACTTGATTTGACACTTCTTT
RSDN-F	GAACTGGGTTACCCGGGGCACCTGTC
RSDN-R	ACTTCTTGTTGTTGACGCTAACATTCAACGCTAGTATTTCCTCTAATCAGGTTCCACC
RSPPDS-F	AACTTAGTTTCGAATAAACACACATAAACAAACAAAAATGGCAGCCGCTATGGTTTTGT
RSPPDS-R	TAAAAAAAAAAAATCTTTGACTATTCAATCATTGCGCTCAGTTGTGTGGGTGTAAGTGG
RSTDH3-F	CCGGGGTATCTGTTTGGTGGAACCTGATTAGAGGAA...
RSTDH3-R	GGACAATGAAAAGAACAAAACCATAGCGGCTGCCATTTTTGTTTGTTTATGTGTGTTTA
RSUP-F	CCAGGTTAACCTGCATTAATGAATCGGCCAACGCATGAGAGTAGCAAACGTAAGTCTAA
RSUP-R	CTCACTATTTTTTACTGCGGAAGCGG
RSURA-F	ATAAAAAAAAAAAGAAGTGTCAAATCAAGTGTCAAATCAGCTGAAGCTTCGTACGCTGC
RSURA-R	ATAAAACAACCTTTAGACTTACGTTTGCTACTCTCATGCGTTGGCCGATTCATTAATGC
21DN-F	AGACTGTCAAGGAGGGTATTCTGGGCCTCCATGTCGCTGAACAGGCATGGGAAGATTCG
21DN-R	TCTGGTGAGGATTTACGGTATG
21GT45-F	AACTTAGTTTCGAATAAACACACATAAACAAACAAAATGGAGAGAGAAATGTTGAGCAA
21GT45-R	GTCTCCCGTCTTCTGTTTAATGATGATGGTGGTGGTGTCAGGAGGAAACAAGCTTTGAA
21KAN-F	CATTATGCAACGCTTCGGAAAATACGATGTTGAAAATTCGACACTGGATGGCGGCGTTA
21KAN-R	AATTCAAAAAAAAAAAGCGAATCTTCCCATGCCTGTTCAGCGACATGGAGGCCCAGAAT
21PRM9-F	AAAGCTTGTTTCCTCCTGACACCACCACCATCATCATTAAACAGAAGACGGGAGACACT
21PRM9-R	CTGTCGATTCGATACTAACGCCGCCATCCAGTGTCGAATTTTCAACATCGTATTTTCCG
21TDH-F	AACACTGGGGCAATAGGCTGTCGCCATTCAAGAGCAGATACTAGCGTTGAATGTTAGCG
21TDH-R	ACATAATGTGAGTTTTGCTCAACATTTCTCTCTCCATTTTGTTTGTTTATGTGTGTTTA
21UP-F	CCCAAAGCTAAGAGTCCCAT
21UP-R	AAACTTCTTGTTGTTGACGCTAACATTCAACGCTAGTATCTGCTCTTGAATGGCGACAG
22DN-F	CTGTCGATTCGATACTAACGCCGCCATCCAGTGTCGAATGAAGCAGGTGTTGTTGTCTG
22DN-R	TCGAGGAGAACTTCTAGTATATTC
22KAN-R	TAAGGCTAACTCTCAACAGACAACAACACCTGCTTCATTCGACACTGGATGGCGGCGTT
22PRM9-F	ACTAGTTAGTAGATGATAGTTGATTTCTATTCCAACAATTTTCAACATCGTATTTTCCG
22UP-F	GGATATAGGAATCCTCAAAATG

22UP-R	CATTATGCAACGCTTCGGAAAATACGATGTTGAAAATTGTTGGAATAGAAATCAACTAT
23KAN-F	TAAAAAAGGAGTAGAAACATTTTGAAGCTATGGCGCGATTATACGAAGTTATCAGCGAC
23UTDH-R	ATTCTGGGCCTCCATGTCGCTGATAACTTCGTATAATCGCGCCATAGCTTCAAAATGTT
24PRM9-F	CCTCAAGCTGGATGATCACCACCACCATCATCATTAAACAGAAGACGGGAGACACTAGC
24TDH3-R	AAACTTTCAATGGTTGTTCAACTTCAACACCTTCCATTTTGTTTGTTTATGTGTGTTTA
25PRM9-F	AGCACAACCTAATAATTGACACCACCACCATCATCATTAAACAGAAGACGGGAGACACT
25TDH3-R	GAAGAGGATAAGATTTATGGGTGATTTCGGAAACCATTTTGTTTGTTTATGTGTGTTTA
2473F3-F	GAACTTAGTTTCGAATAAACACACATAAACAAACAAAATGGAAGGTGTTGAAGTTGAAC
2473F3-R	GTCTCCCGTCTTCTGTTTAATGATGATGGTGGTGGTGATCATCCAGCTTGAGGTCTCTC
2573C10-F	AACTTAGTTTCGAATAAACACACATAAACAAACAAAATGGTTTCCGAAATCACCCATAA
2573C10-R	GTCTCCCGTCTTCTGTTTAATGATGATGGTGGTGGTGTCAATTATTAGGTTGTGCTAGT

Table S3. Simulation information for both systems.

System	Temperature (K)	Time (ns)	Traj. Num.	Ions	Waters
UGTPg45	298	60	1	13 Na^+	12193
UGTPg45-HV	298	60	1	13 Na^+	12179
